# Systematic analysis of various RNA transcripts and construction of biological regulatory networks at the post-transcriptional level for chronic obstructive pulmonary disease

**DOI:** 10.1186/s12967-023-04674-7

**Published:** 2023-11-07

**Authors:** Beibei Li, Jiajun Zhang, Hui Dong, Xueyan Feng, Liang Yu, Jinyuan Zhu, Jin Zhang

**Affiliations:** 1 School of Clinical Medicine, Ningxia Medical University, Yinchuan, 750004 China; 2 Center of Research Equipment Management, General Hospital of Ningxia Medical University, Yinchuan, 750004 China; 3 Department of Thoracic Surgery, General Hospital of Ningxia Medical University, Yinchuan, 750004 China; 4 Department of Critical Care Medicine, General Hospital of Ningxia Medical University, Yinchuan, 750004 China; 5 Department of Respiratory and Critical Care Medicine, General Hospital of Ningxia Medical University, 804 Shengli South Street, Xingqing District, Yinchuan, 750004 China

**Keywords:** COPD, Hub gene, RBP, ceRNA network, Immune cell infiltration, Therapeutic target

## Abstract

**Background:**

Although chronic inflammation, oxidative stress, airway remodeling, and protease-antiprotease imbalance have been implicated in chronic obstructive pulmonary disease (COPD), the exact pathogenesis is still obscure. Gene transcription and post-transcriptional regulation have been taken into account as key regulators of COPD occurrence and development. Identifying the hub genes and constructing biological regulatory networks at the post-transcriptional level will help extend current knowledge on COPD pathogenesis and develop potential drugs.

**Methods:**

All lung tissues from non-smokers (n = 6), smokers without COPD (smokers, n = 7), and smokers with COPD (COPD, n = 7) were collected to detect messenger RNA (mRNA), microRNA (miRNA), circular RNA (circRNA), and long non-coding RNA (lncRNA) expression and identify the hub genes. Biological regulatory networks were constructed at the post-transcriptional level, including the RNA-binding protein (RBP)-hub gene interaction network and the competitive endogenous RNA (ceRNA) network. In addition, we assessed the composition and abundance of immune cells in COPD lung tissue and predicted potential therapeutic drugs for COPD. Finally, the hub genes were confirmed at both the RNA and protein levels.

**Results:**

Among the 20 participants, a total of 121169 mRNA transcripts, 1871 miRNA transcripts, 4244 circRNA transcripts, and 122130 lncRNA transcripts were detected. There were differences in the expression of 1561 mRNAs, 48 miRNAs, 33 circRNAs, and 545 lncRNAs between smokers and non-smokers, as well as 1289 mRNAs, 69 miRNAs, 32 circRNAs, and 433 lncRNAs between smokers and COPD patients. 18 hub genes were identified in COPD. TGF-β signaling and Wnt/β-catenin signaling may be involved in the development of COPD. Furthermore, the circRNA/lncRNA-miRNA-mRNA ceRNA networks and the RBP-hub gene interaction network were also constructed. Analysis of the immune cell infiltration level revealed that M2 macrophages and activated NK cells were increased in COPD lung tissues. Finally, we identified that the ITK inhibitor and oxybutynin chloride may be effective in treating COPD.

**Conclusions:**

We identified several novel hub genes involved in COPD pathogenesis. TGF-β signaling and Wnt/β-catenin signaling were the most dysregulated pathways in COPD patients. Our study constructed post-transcriptional biological regulatory networks and predicted small-molecule drugs for the treatment of COPD, which enhanced the existing understanding of COPD pathogenesis and suggested an innovative direction for the therapeutic intervention of the disease.

**Supplementary Information:**

The online version contains supplementary material available at 10.1186/s12967-023-04674-7.

## Background

Chronic obstructive pulmonary disease (COPD) is a typical chronic and progressive respiratory disease characterized by irreversible airflow limitation and recurrent respiratory symptoms. The inflammatory response, oxidative stress, protease-antiprotease imbalance, chronic tissue damage and repair, as well as associated genes and genetic signals, all play a role in COPD pathogenesis [1]. Smoking is the primary cause of compromised pulmonary function stemming from COPD pathogenesis. Smoking-induced oxidative stress and excessive protease synthesis can result in alveolar epithelial cell death, degradation of the extracellular matrix, and loss of alveolar structural integrity [2]. Despite great advances in the study of COPD, there are still no effective drugs to control or delay the progression of the disease. Therefore, it is necessary to have an improved understanding of the molecular mechanism of the disease to identify molecular targets so as to achieve the development of innovative drugs.

High-throughput sequencing has greatly increased our knowledge of the genes, molecular processes, and pathways of many human diseases. Advances in bioinformatics have enhanced our comprehension of disease pathogenesis and contributed to the investigation of therapeutic targets [3]. Although previous studies of biomarkers of COPD in smokers and non-smokers have revealed hundreds of mRNAs associated with the potential pathogenesis, the correspondence is not always linear between transcript level and protein abundance [4–7]. These results suggest that single transcriptomic data might not completely elucidate COPD, and new research ideas are urgently needed, such as constructing biological regulatory networks according to post-transcriptional regulatory mechanisms. Two components are important for post-transcriptional mechanisms: non-coding RNAs and RNA-binding proteins (RBPs). Non-coding RNAs comprise microRNA (miRNA), long non-coding RNA (lncRNA), and circular RNA (circRNA). Many studies have demonstrated that non-coding RNA can target mRNA through the competing endogenous RNA (ceRNA) mechanism, regulate different biological functions involving inflammation, apoptosis, proliferation, and epithelial-mesenchymal transition (EMT), and contribute to the occurrence and development of COPD [8–10]. RBPs regulate the expression of genes associated with cell proliferation, oxidative stress, apoptosis, and immune cell skewing by combining with conserved RNA-binding domains [11].

Peripheral blood is a commonly available biomarker for histology studies, but it is susceptible to the environment and comorbidities. Lung tissue is relatively stable, can store more information, and provides more insight into the mechanisms of COPD. To our knowledge, there are few studies about the systematic analysis of the molecular mechanism of COPD by integrating the whole transcriptome-sequencing (mRNA and non-coding RNA) data of lung tissue. By analyzing the whole-transcriptome sequencing data of lung tissues from non-smokers, smokers, and COPD patients, in this study, we intended to: (1) describe the changes in mRNA and non-coding RNA expression profiles in smokers and COPD patients and identify hub genes; (2) analyze the function, molecular mechanism, and pathway of differential mRNAs; (3) integrate the RBP-hub gene and circRNA/lncRNA-miRNA-mRNA biological regulatory networks to reveal the molecular mechanism of regulating COPD-related mRNAs at the post-transcriptional level; and (4) predict potential therapeutic drugs for COPD combined with mRNA expression profiles. Finally, these findings were confirmed at both the RNA and protein levels by quantitative real-time PCR and western blot analysis. We believe this study will advance our knowledge of COPD pathogenesis and offer novel perspectives on medical treatments for this disease.

## Methods

### Patients and specimens

Lung tissue specimens were collected from 20 patients undergoing lobectomy in the General Hospital of Ningxia Medical University. The specimens were normal lung tissues at least 5 cm away from the lesions, according to previous reports [12, 13]. The clinical characteristics of these patients are listed in Table 1. All lung specimens were divided into three groups: non-smokers (NS, n = 6), current smokers without COPD (smokers, SM, n = 7), and current smokers with COPD (COPD, n = 7). Non-smokers are people who smoke fewer than 100 cigarettes in their lifetime or never smoke a cigarette. Smokers are people who quit smoking during the 12 months before the experiment or are currently smoking. COPD was diagnosed in accordance with the Global Initiative for Chronic Obstructive Lung Disease criteria. None of these patients had received any chemotherapy or radiation therapy before surgery. COPD patients had received only bronchodilators for 3 months prior to the surgery, and none had ever received any corticosteroids or antibiotics. In addition, patients with asthma, pulmonary infections, and other respiratory diseases were also excluded. All participants underwent multi-slice spiral computed tomography (CT) scans. Images were analyzed using 3D Slicer (v5.2.2) software. In this study, the degree of emphysema and airway wall thickness were evaluated by calculating %LAA_-950_ and Pi10 [14, 15]. %LAA_-950_ was defined as the percentage of voxels with attenuation lower than -950 Hounsfield units (%LAA_-950_). Pi10 was defined as the square root of the wall area of a hypothetical airway with an internal perimeter of 10 mm (Pi10). Ethical approval for the study was granted by the Ethics Committee of the General Hospital of Ningxia Medical University. Written informed consent was obtained from each patient. The study complies with the Declaration of Helsinki.

**Table 1:** Information about the participants in this study

	Non-smokers (n = 6)	Smokers without COPD (n = 7)	Smokers with COPD (n = 7)
Gender (F/M)	5/1	0/7	0/7
Age(years)	53.83 ± 6.59	55.43 ± 11.59	60.14 ± 11.82
Smoking history(pack-years)	0	23.33 ± 10.84**	38.93 ± 21.24**
FEV1/FVC%	80.50 (79.25, 81)	77 (75, 77.50)*	65.34 (63, 67)**##
FEV1(%predicted)	96.17 ± 1.83	95.89 ± 5.31	78.57 ± 5.06**##
BMI (kg/m^2^)	23.75 (23.41, 24.04)	25.51 (24.51, 26.97)	24.97 (24.12, 25.85)
Disease constitution			
Lung adenocarcinoma	5	5	2
Lung squamous cell carcinoma	0	0	4
Lung benign nodules	1	2	1
Quantitative CT measurements			
%LAA_-950_	0.37 ± 0.23	0.52 ± 0.83	3.03 ± 1.87#
Pi10	5.69 ± 0.54	5.97 ± 0.12	6.48 ± 0.97

*BMI* body mass index, *FEV1* forced expiratory volume in 1 s, *FVC* forced vital capacity

^*^P < 0.05, **P < 0.01, different from non-smokers; #P < 0.05, ##P < 0.01 different from smokers

### High-throughput sequencing

Total RNA was prepared from human lung tissues with the TRIzol reagent (Invitrogen, USA). For total RNA samples, purity and integrity were assessed with an Agilent 2100 Bioanalyzer (Agilent, USA). The quality control parameters are RIN ≥ 7 and 28S/18S ratio ≥ 1.5. Ribosomal RNA was depleted from total RNA with the Ribo-Zero™ Magnetic Kit (Epicentre, USA). The TruSeq™ Small RNA Sample Prep Kit (Illumina, USA) and the NEBNext Ultra™ RNA Library Prep Kit for Illumina (NEB, USA) were used for constructing sequencing libraries for miRNA, circRNA, lncRNA, and mRNA, respectively. Subsequently, the libraries were sequenced with the Illumina NovaSeq 6000 system (CapitalBio Technology, China). The original image files of Illumina high-throughput sequencing were identified by bcl2fastq (v2.17.1.14) software and converted into the original sequencing sequences. The set of original sequencing sequences constituted raw data. Sequencing quality was determined using the FastQC (v0.11.2) software. The fastp (v0.14.0), cutadapt (v1.7.1), and FASTX toolkit (v0.0.14) software were applied to filter the raw data and acquire clean reads. The alignment of clean reads to the reference genome sequence (Homo sapiens version GRCh38) was performed through the TopHat (v2.0.13) and HISAT2 (v2.1.0) software, which is the basis for all subsequent analyses. The expression of mRNA and lncRNA was normalized to FPKM, while the normalized TPM and SRPBM represented the expression levels of miRNA and circRNA, respectively. A mean transcript expression of no less than 0.5 was used for the differentially expressed gene analysis.

### Identification of RNA transcripts with differential expression

The limma (v3.32.10) package in R software was employed to identify the mRNAs, miRNAs, circRNAs, and lncRNAs that were differentially expressed between non-smokers and smokers, as well as smokers and COPD patients. The screening criteria were a P value < 0.05 and log2 (fold change) ≥ 1 (upregulated) or log2 (fold change) ≤ − 1 (downregulated). The UpSet plot was generated with UpSetR (v1.4.0) and yyplot (v0.0.8) packages in R software to visualize the differential mRNAs.

### Identification of hub genes

To identify hub genes, we upload the differentially expressed mRNAs to the Search Tool for the Retrieval of Interacting Genes (STRING, v11.5). The disconnected nodes were hidden in the network, with the minimum required interaction score set to 0.4. The obtained networks were uploaded to the Cytoscape (v3.0.1) software. The MCODE plug-in was used to identify hub genes.

### Gene Ontology (GO) functional annotation and pathway enrichment analysis

Gene Ontology biological process (GO_BP), and Kyoto Encyclopedia of Genes and Genomes (KEGG) pathway enrichment analysis were carried out with the clusterProfiler (v3.14.3) and GOplot (v1.0.2) packages in R software as well as the DAVID online database (https://david.ncifcrf.gov, v2022q2). Pathway and process enrichment analysis of hub genes was carried out with the Metascape online database (http://metascape.org/gp/index.html#/main/step1). The visualization of results from enrichment analysis was performed by Bioinformatics (https://www.bioinformatics.com.cn), a free online platform for data analysis and visualization.

### Gene set enrichment analysis (GSEA)

By GO/KEGG analysis, the biological pathways of up- and down-regulated gene enrichment can be identified, but it may miss genes with important biological significance that are not significantly differentially expressed, ignoring some valuable information such as the biological characteristics of genes, the relationship between gene regulatory networks, and gene function and significance. GSEA analysis is based on the analysis of all gene expression data, focusing on the expression pattern of the entire gene set. It considers not only the individual genes with differential expression but also the enrichment of the whole gene set. At present, it is generally combined with the results of the two analysis methods to make inferences. Gene set enrichment analysis was performed via the GSEA (Broad Institute, v4.1.0) software using Hallmarks Sets (hall.all.v7.5.1symbols.gmt) from MsigDB. Gene sets enriched with false discovery rate (FDR) q values < 0.25, nominal P values < 0.05, and the |normalized enrichment score (NES)|> 1 were considered to be significant. The enrichment analysis results were visualized with the Bioinformatics.

### Construction of the RBP-hub gene interaction network and the ceRNA network

The target RBPs of the hub genes were obtained from the ENCORI online database (https://starbase.sysu.edu.cn/index.php). The visualization of the RBP-hub gene interaction network was undertaken by Cytoscape (v3.0.1) software. The differentially expressed miRNAs were viewed as the central component for constructing the ceRNA regulatory network. Firstly, the online databases miRDB (http://www.mirdb.org), miRTarBase (http://mirtarbase.mbc.nctu.edu.tw), and miRWalk (http://mirwalk.umm.uni-heidelberg.de) were employed to identify the target mRNAs of differentially expressed miRNAs, constructing the miRNA–mRNA network. Subsequently, the miRanda (v3.3a) software and the miRcode online database (http://www.mircode.org) were applied to predict the target lncRNAs and circRNAs of differentially expressed miRNAs, constructing the circRNA–miRNA network and the lncRNA–miRNA network, respectively. Finally, the circRNA–miRNA network, lncRNA–miRNA network, and miRNA–mRNA network were linked by Cytoscape (v3.0.1) software to construct the circRNA–miRNA–mRNA and lncRNA–miRNA–mRNA networks.

### Evaluation of tissue-infiltrating immune cells

The difference in immune cell infiltration among smokers and COPD patients’ lung tissues was assessed via the CIBERSORTx website (https://cibersortx.stanford.edu). The histograms were plotted using the ggplot2 (v3.3.3), stats (v4.2.1), and car (3.1-0) packages in R software to display the distribution and abundance of 22 immune cell infiltrations.

### Screening of small-molecule therapeutic drugs

The top 150 upregulated and top 150 downregulated genes from COPD were used for drug prediction in the Connectivity Map database (Cmap, v1.1.1.43). Cmap is a comprehensive expression profile database of drug interference treatments and is commonly applied to explore small-molecule drugs for treating diseases. Negative connection scores are considered to represent potential small-molecule therapeutic drugs. PubChem (https://pubchem.ncbi.nlm.nih.gov) was applied to show the molecular structure of the two drugs with the higher negative connection scores.

### Establishment of the COPD rat models

Male Sprague–Dawley rats (6–7 weeks old, 285 ± 15 g) were purchased from SJA (Changsha, China). After a one-week adaptive raise, these rats were randomly assigned to two groups: control (6 rats) and COPD (6 rats). COPD rats were exposed to cigarette smoke (CS) from Hongmei brand cigarettes (Hongta Group, China; 0.8 mg nicotine and 10 mg tar per cigarette). This model was established according to previously reported methods, with minor modifications [16–18]. Briefly, rats were exposed to CS in the whole-body smoke exposure system (Guangdong Huawei Testing Co. Ltd., Guangdong, China). These rats were exposed to CS for 40–60 min, separated by a break of 60 min, four sessions per day, five days per week, for up to 16 weeks. Age- and weight-matched male rats exposed to the air were used as controls. All rats ate and drank freely under the same conditions, including a 12-h diurnal rhythm, a relative humidity of 40–70%, and a temperature of 21–23 °C. All rats received humane care in accordance with the 3R principles for experimental animals throughout the experiment.

### Evaluation of the COPD rat models

All rats were weighed 24 h after the last exposure. Then, the rats were intubated under general anesthesia, and pulmonary function tests were assessed using the Buxco FinePointe Pulmonary Function Test system (Data Sciences International). The bronchoalveolar lavage fluid (BALF) was obtained by perfusing the right lung three times with 6 ml of saline through a BALF cannula inserted into the trachea. A hemocytometer was used to quantify the leukocyte count in the BALF, and then Wright-Giemsa staining was done to identify the BALF cells. The right lung tissue was snap-frozen and stored for RNA analysis. The left lung was perfused with and fixed overnight in 4% paraformaldehyde and processed for paraffin embedding. Samples were processed into 4 μm sections on a microtome for subsequent hematoxylin and eosin (H&E) staining. The stained slides were entirely scanned by the Pannoramic MIDI automatic digital slide scanner (3DHISTECH, Hungary) and processed with CaseViewer (v2.4) software. For the morphometric assessment of emphysema and airway remodeling, the mean linear intercept (MLI), mean alveolar number (MAN), and small airway wall thickness were calculated in H&E-stained sections of rat lung tissues. The MLI, a measurement of the mean distance between alveolar walls, was calculated as the count of intersected alveolar walls per unit length of the control line. The MAN, a measurement of alveolar density, was calculated as the count of alveoli per unit area. Regular small bronchial sections with an inner circumference < 1000 μm were randomly selected, and the area of the airway wall was normalized by the length of the basement membrane to define the airway wall thickness. The polygon tool in CaseViewer was applied to measure the external airway area (external area) and internal airway area (internal area), as well as the length of the airway circumference (circumference). The difference between the external and internal areas was divided by the “circumference” to derive the airway wall thickness.

### Reverse transcription and real-time quantitative PCR

Total RNA was prepared from rat lung tissues with the TRIzol reagent (Invitrogen, USA) and generated into cDNA with the RevertAid First Strand cDNA Synthesis Kit (Thermo Scientific, USA). Quantitative real-time PCR reactions were run on the LightCycler^®^ 480II System (Roche, Switzerland) with the TB Green^®^ Premix Ex Taq™II (Tli RNaseH Plus) kit (Takara Bio, Japan). The β-actin gene served as an internal reference for normalization. All primers used were purchased from Sangon Biotech (Shanghai, China), and the sequences are provided in Additional file 1: Table S1.

### Western blot analysis

Total protein from lung tissue samples was prepared with RIPA lysis buffer (Thermo, USA) and quantified using the BCA Protein Assay Kit (KeyGene, China). Equal amounts of proteins were separated on SDS–polyacrylamide gel electrophoresis (PAGE) and transferred onto polyvinylidene fluoride (PVDF) membranes (Millipore, USA). After blocking with 5% skim milk for 1.5 h at room temperature, the membranes were incubated with primary antibodies against DDB2 (Proteintech, 10431-1-AP), RUVBL1 (Proteintech, 10210-2-AP), and GAPDH (Abcam, ab181602) overnight at 4 °C, followed by incubation with HRP-conjugated goat anti-rabbit secondary antibodies (ABclonal, AS014) for 1.5 h at room temperature. Protein bands were visualized using the enhanced chemiluminescence system (Invigentech, USA). The chemiluminescent signals were detected using ImageQuant 800 (GE Healthcare) and quantified with the Gel-Pro analyzer software (Media Cybernetics Inc., v4.0). Raw data for western blot can be found in Additional file 2.

### Double immunofluorescence staining of lung tissue

In this study, we used rabbit anti-CD16 antibody (Proteintech, 16559-1-AP) and mouse anti-CD56 antibody (Proteintech, 60238-1-Ig) to label activated NK cells, while rabbit anti-CD68 antibody (Proteintech, 25747-1-AP) and mouse anti-CD206 antibody (Proteintech, 60143-1-Ig) were used to identify M2 macrophages in human lung tissues. Before the double immunofluorescence staining, paraffin-embedded tissues were sectioned at 4 μm thickness. Heat-mediated antigen retrieval was conducted with EDTA buffer (PH 9.0). Tissue sections were then blocked with 10% goat serum (ZSGB-BIO, SAP-9100) for 30 min at room temperature. For the double immunofluorescence staining, the sections were incubated with a mixture of primary antibodies overnight at 4 °C in a humidified chamber. After washing in cold PBS, sections were incubated with a mixture of FITC-conjugated goat anti-rabbit (Abbkine, A22120) and Dylight 594-conjugated goat anti-mouse (Abbkine, A23410) secondary antibodies for 30 min at room temperature in the dark. Nuclei were counterstained with DAPI (G-CLONE, PN4311). Stained sections were scanned by the Pannoramic MIDI automatic digital slide scanner (3DHISTECH, Hungary) and processed with CaseViewer (v2.4) software. The number of positive cells was quantified using Image J (v1.51) software.

### Statistical analysis

We used R (v3.6.3) software for all statistical analyses. All experimental data were presented as mean ± standard deviations. Comparisons between two groups were performed with the Student’s t test, and comparisons among multiple groups were assessed by one-way analysis of variance (ANOVA). P values < 0.05 were considered to be significantly different.

## Results

### Identification of differentially expressed RNA transcripts

**Fig. 1:**
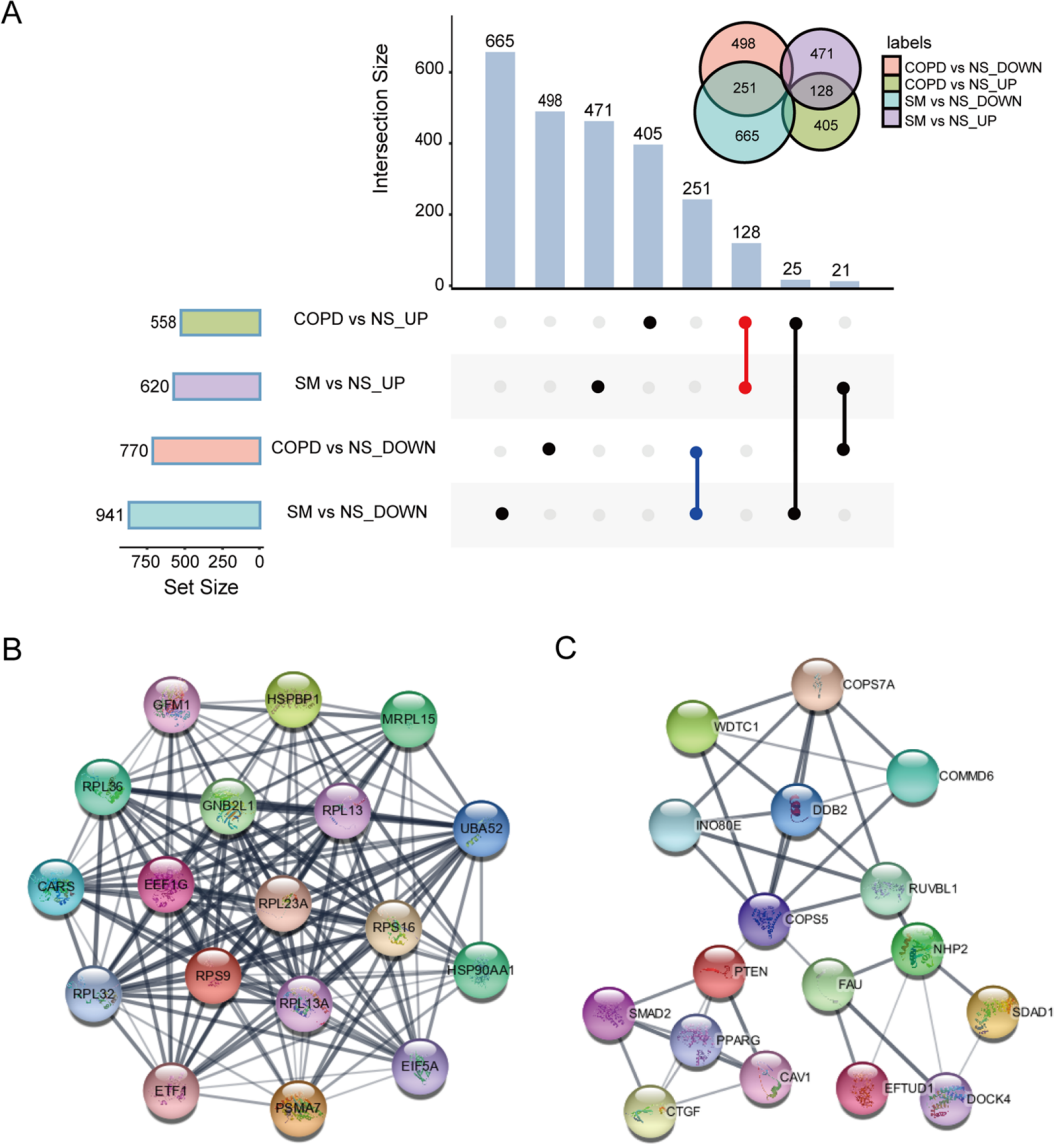
Analysis of mRNA expression profiles and identification of hub genes. **A** The Upset plot shows the number of unique and shared differentially expressed mRNAs in smokers and COPD patients. **B** Hub genes in COPD patients. **C** Hub genes shared by smokers and COPD patients

To identify the RNAs associated with COPD, we performed whole-transcriptome sequencing of lung tissues from six non-smokers, seven smokers, and seven COPD patients. Among the 20 participants, a total of 121169 mRNA transcripts, 1871 miRNA transcripts, 4244 circRNA transcripts, and 122130 lncRNA transcripts were detected. After a differential analysis, there were differences in the expression of 1561 mRNAs, 48 miRNAs, 33 circRNAs, and 545 lncRNAs between smokers and non-smokers (Additional file 1: Tables S2–S5), as well as 1289 mRNAs, 69 miRNAs, 32 circRNAs, and 433 lncRNAs between smokers and COPD patients (Additional file 1: Tables S6–S9). Additionally, in comparison to non-smokers, the Upset plot showed 379 genes (128 upregulated and 251 downregulated) with the same trend of expression in smokers and COPD patients (Fig. 1A).

### Validation of hub genes

Compared with smokers, 18 hub genes were identified in COPD patients, including GFM1, HSPBP1, MRPL15, RPL36, GNB2L1, RPL13, UBA52, CARS, EEF1G, RPL23A, RPS16, HSP90AA1, RPL32, RPS9, RPL13A, EIF5A, ETF1, and PSMA7 (Fig. 1B). Meanwhile, compared with non-smokers, 17 hub genes were identified in differentially expressed mRNAs shared by smokers and COPD patients, including COPS7A, WDTC1, COMMD6, DDB2, INO80E, RUVBL1, COPS5, PTEN, SMAD2, PPARG, CAV1, CTGF, FAU, NHP2, SDAD1, EFTUD1, and DOCK4 (Fig. 1C), which may increase the risk of COPD in people with a history of smoking.

### Evaluation of the COPD rat models and validation of the hub genes

**Fig. 2:**
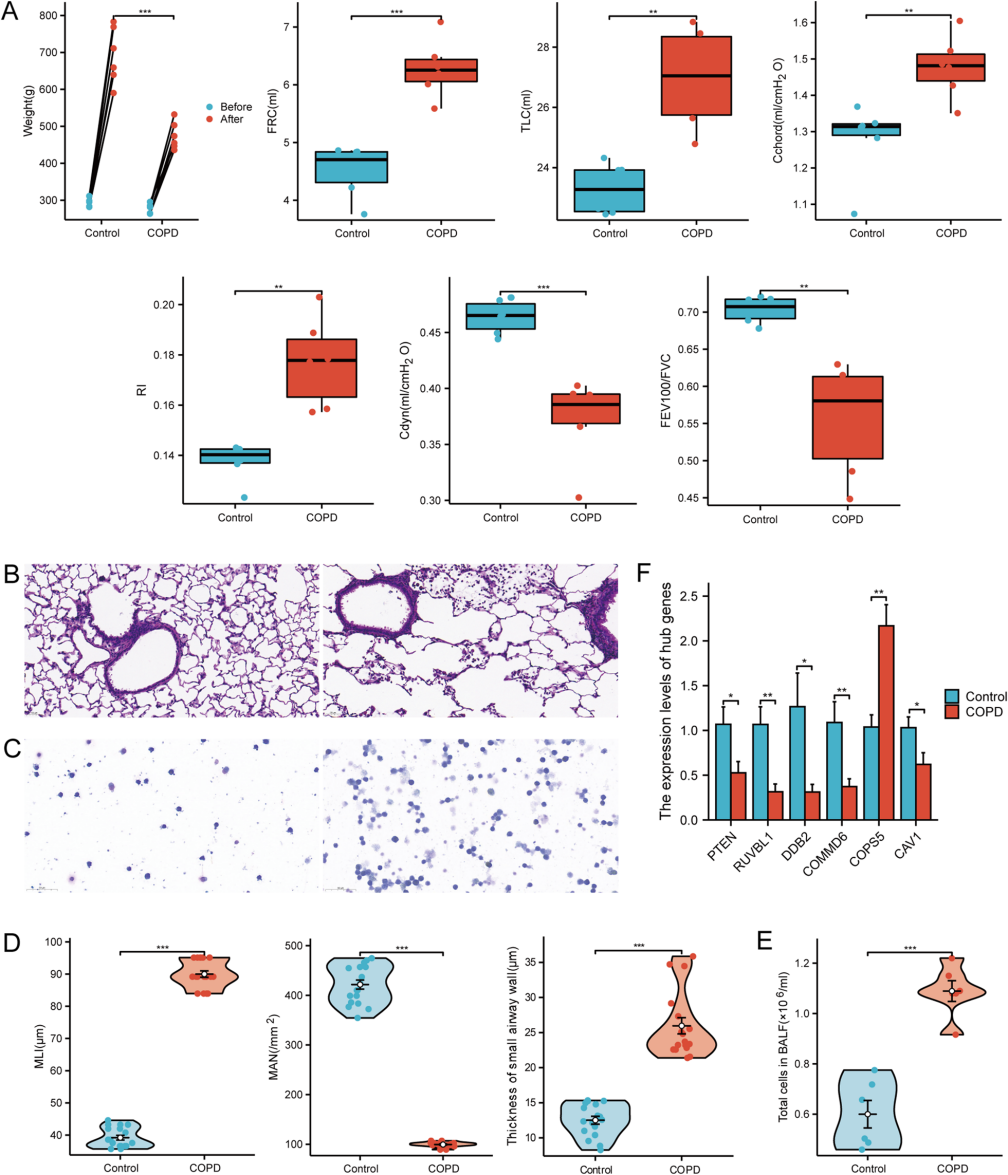
Evaluation of the COPD rat models and validation of the hub genes at the RNA level. **A** Body weight and lung function of COPD rats and controls. **B** Representative images of H&E staining of lung tissue in COPD rats (right panel) and controls (left panel). **C** Representative images of Wright-Giemsa staining of BALF cells in COPD rats (right panel) and controls (left panel). **D** Morphometric measurements of MLI, MAN, and small airway walls in lung tissue sections with H&E staining. **E** BALF cell counts. **F** Validation of hub genes by qRT-PCR in COPD rat models and controls. Scale bar: 50 μm. *P < 0.05, **P < 0.01, ***P < 0.001

**Fig. 3:**
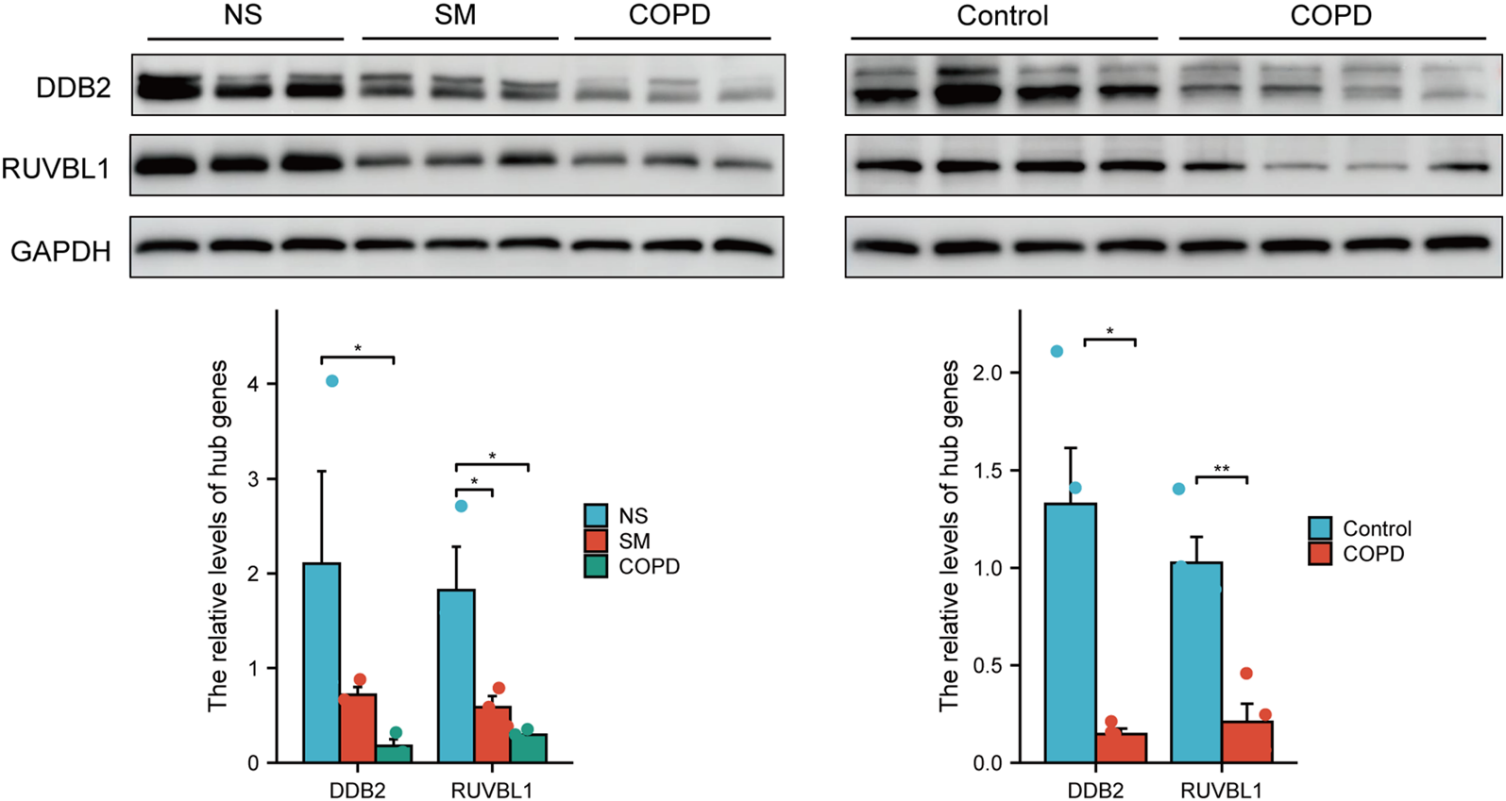
Validation of hub genes at the protein level. Western blot analysis shows DDB2 and RUVBL1 protein expression in lung tissues from non-smokers, smokers without COPD, and smokers with COPD (upper left panel and lower left panel), as well as COPD rats and controls (upper right panel and lower right panel). *P < 0.05, **P < 0.01

To validate the differentially expressed mRNAs from whole-transcriptome sequencing, we established CS-exposed COPD rat models. We found that CS-exposed rats exhibited typical COPD-like lung function decline compared with controls (Fig. 2A), manifested as increased functional residual capacity (FRC), total lung capacity (TLC), chord compliance (Cchord), resistance (RI), as well as decreased dynamic compliance (Cdyn) and the FEV100/FVC ratio. H&E staining analysis demonstrated that the alveolar structure of CS-exposed rats was disorganized, the alveolar wall became thinner or ruptured, and it fused into bullae (Fig. 2B). MAN, MLI, and airway wall thickness were further employed to assess emphysema severity and airway remodeling in rats. The results revealed a significantly thickened small airway wall and a markedly higher MLI with a decrease in MAN in CS-exposed rats (Fig. 2D). CS exposure causes airway and lung inflammation, manifested by a higher leukocyte count in BALF (Fig. 2C, E). Emaciation is an indicator of the severity of COPD. The body weight of rats exposed to CS was significantly lower than that of rats exposed to air (Fig. 2A). These data suggest that our CS-exposed rats exhibit the chronic disease characteristics of human COPD disease. We verified the mRNA levels of six hub genes in rat lung tissues (Fig. 2F) and further verified the protein levels of two hub genes in both human and rat lung tissues (Fig. 3). Findings from q-PCR and western blot were consistent with the sequencing results, indicating the high accuracy of our sequencing results.

### GO functional annotation and pathway enrichment analysis

**Fig. 4:**
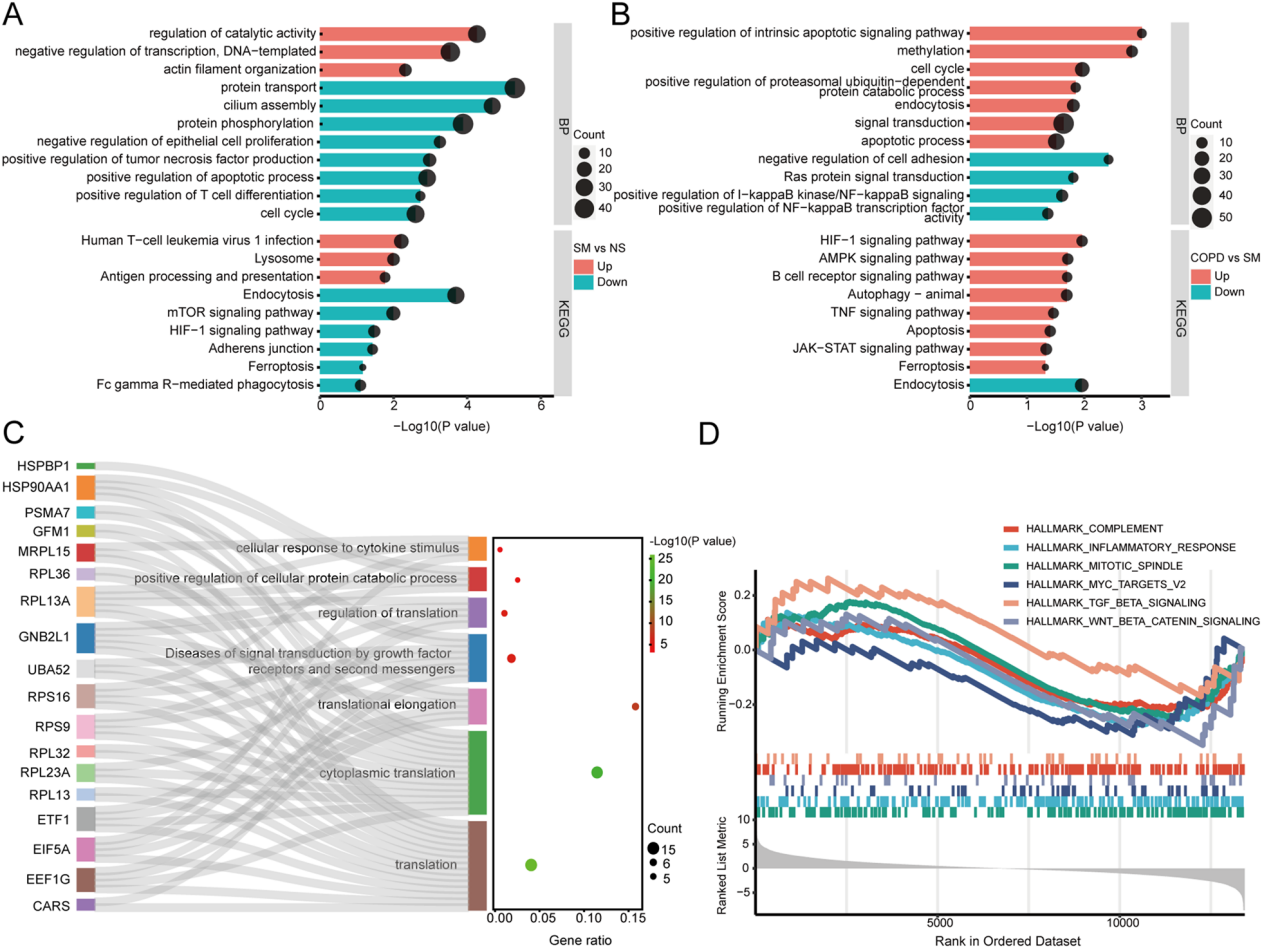
GO functional annotation and pathway enrichment analysis. **A** GO_BP annotation and KEGG enrichment analysis of differentially expressed mRNAs between non-smokers and smokers. **B** GO_BP annotation and KEGG enrichment analysis of differentially expressed mRNAs between smokers and COPD patients. **C** Pathway and process enrichment analysis of hub genes between smokers and COPD patients. **D** GSEA enrichment analysis of gene expression data in smokers and COPD patients

Exploring the functions and signaling pathways involved in genes is generally beneficial for investigating the molecular mechanisms of disease. We carried out GO and KEGG enrichment analyses on mRNAs with differential expression. Compared with non-smokers, the upregulated mRNAs in smokers were primarily enriched in human T-cell leukemia virus 1 infection, the lysosome, and antigen processing and presentation, and the downregulated mRNAs were enriched in endocytosis, the mTOR and HIF-1 signaling pathways, adherens junction, ferroptosis, and Fc gamma R-mediated phagocytosis (Fig. 4A). Whereas compared with smokers, the upregulated mRNAs in COPD were primarily enriched in the HIF-1 and AMPK signaling pathways, the B cell receptor signaling pathway, autophagy-animal, the TNF signaling pathway, apoptosis, the JAK-STAT signaling pathway, and ferroptosis, and the downregulated mRNAs were enriched in endocytosis (Fig. 4B). Meanwhile, we also performed GO and KEGG enrichment analyses of mRNAs with differential expression shared by smokers and COPD patients (Additional file 2: Fig. S1). Additionally, we utilized Metascape to carry out pathway and process enrichment analysis on the hub genes in COPD (Fig. 4C) as well as on the hub genes shared by smokers and COPD patients (Additional file 2: Fig. S2). GSEA enrichment analysis of gene expression data in smokers and COPD patients indicated that COPD was significantly associated with the mitotic spindle, inflammatory response, myc targets, complement, TGF-β, and Wnt/β-catenin pathways (Fig. 4D; Additional file 1: Table S10).

### Construction of the RBP-hub gene interaction network and the ceRNA network

**Fig. 5:**
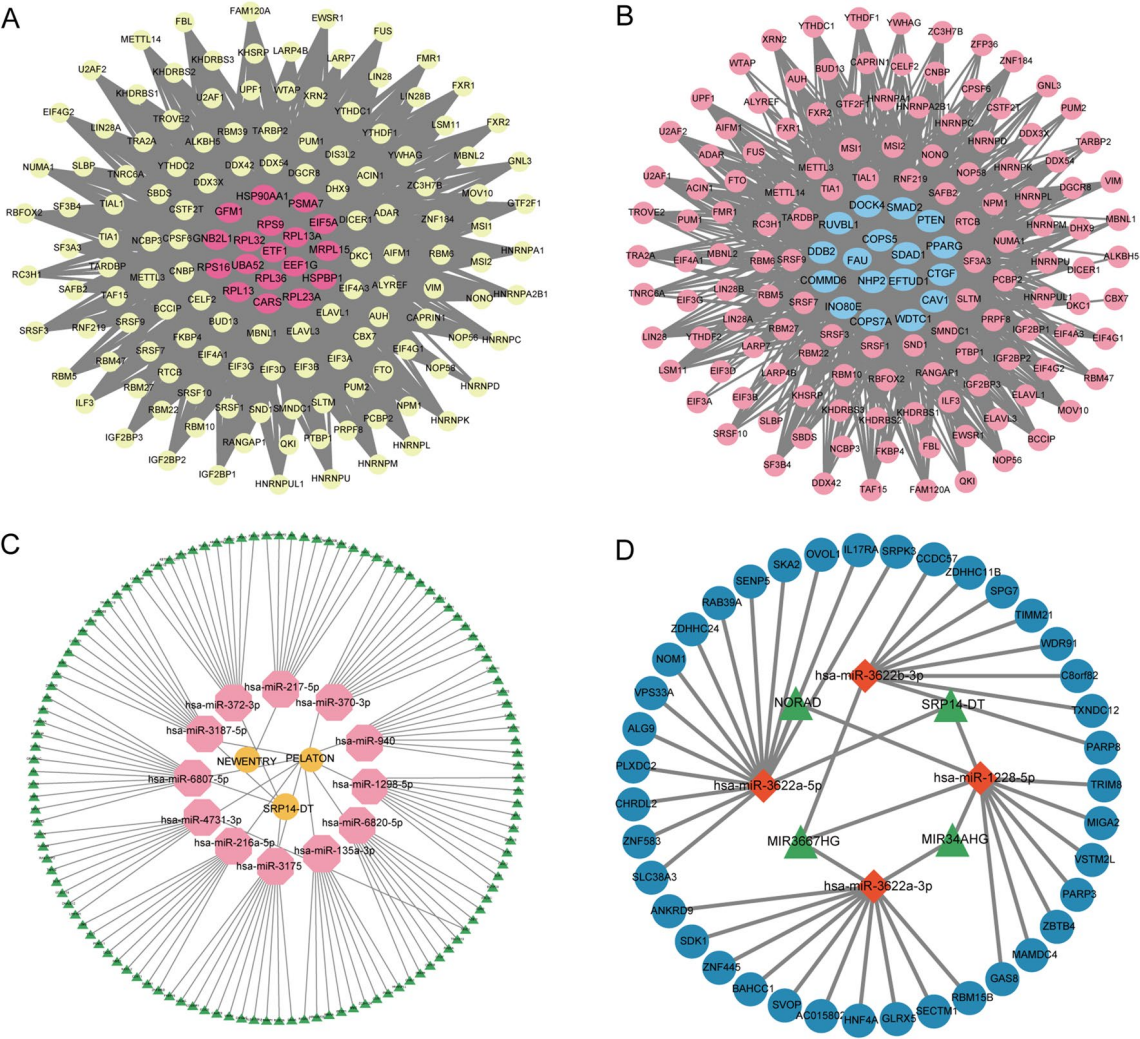
RBP-hub gene interaction network and the ceRNA network. **A** RBP-hub gene interaction network constructed by hub genes in COPD patients. Red nodes in the network represent the hub genes, while yellow nodes represent the corresponding RBPs. **B** RBP-hub gene interaction network constructed by the hub genes shared by smokers and COPD patients. Blue nodes in the network represent the hub genes, while red nodes represent the corresponding RBPs. Connections between different colored nodes indicate that RBPs may regulate hub gene expression by binding to specific RNA structural motifs or elements. **C** Differentially expressed lncRNA-miRNA-mRNA network in COPD patients. **D** Differentially expressed lncRNA-miRNA-mRNA network shared by smokers and COPD patients

We utilized the ENCORI online database and Cytoscape software to construct the RBP-hub gene network and visualize the results. The RBP-hub gene network in COPD patients included 18 hub genes and 130 RBPs (Fig. 5A), while the RBP-hub gene network shared by smokers and COPD patients included 17 hub genes and 129 RBPs (Fig. 5B). In addition to the RBP-hub gene interaction network, the lncRNA-miRNA-mRNA networks were constructed according to differentially expressed miRNAs in COPD patients (Fig. 5C) as well as differentially expressed miRNAs shared by smokers and COPD patients (Fig. 5D). Meanwhile, we also constructed circRNA-miRNA-mRNA networks for these differentially expressed miRNAs (Additional file 2: Fig. S3).

### Immune cell infiltration analysis and identification of small-molecule therapeutic drugs

**Fig. 6:**
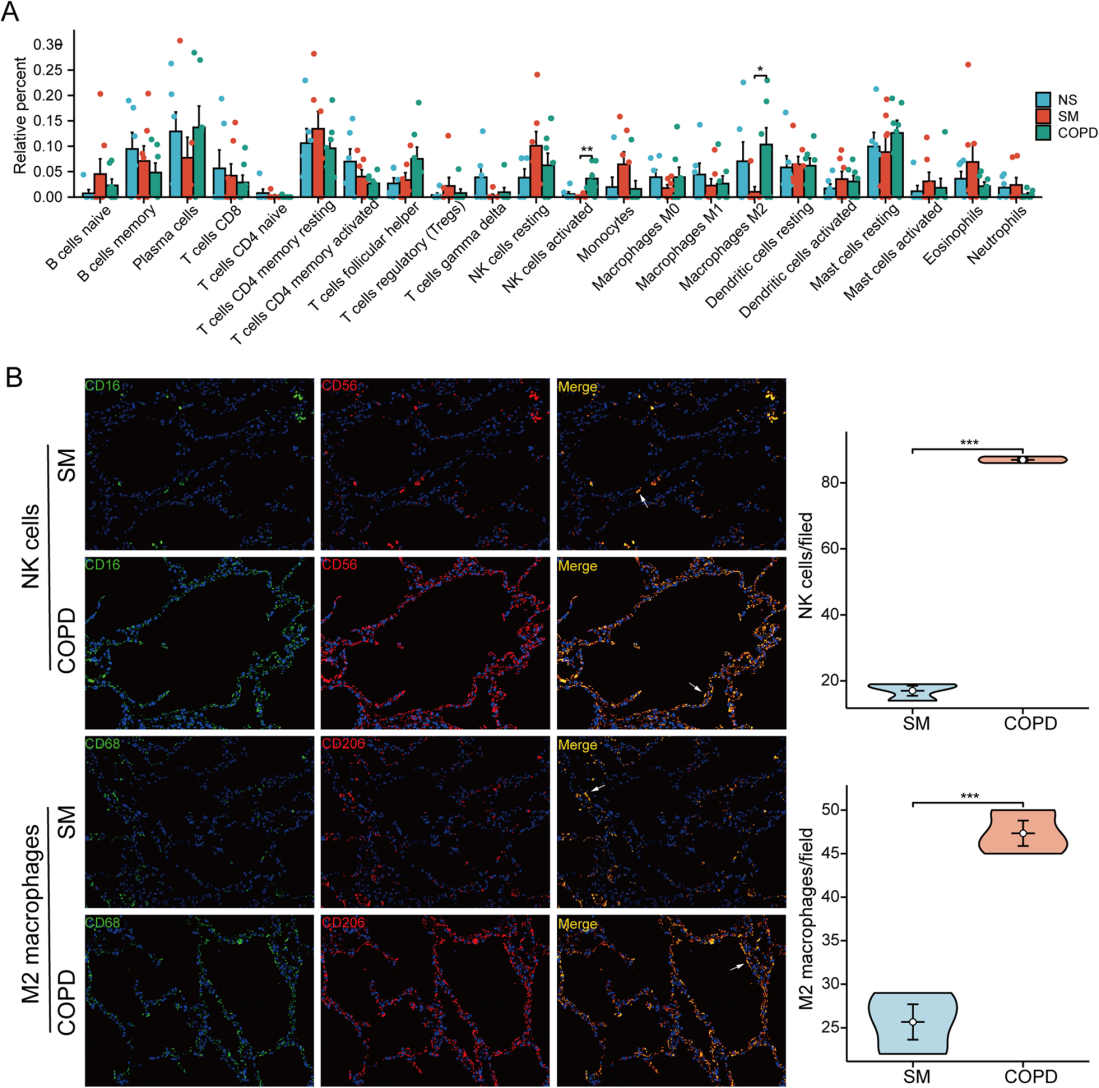
Immune cell infiltration analysis and validation. **A** The composition and abundance of immune cells in lung tissue from non-smokers, smokers, and COPD patients. **B** Double immunofluorescence staining analysis of lung tissues from smokers without COPD and smokers with COPD. Cells with both red and green fluorescence indicate NK cells or M2 macrophages. Scale bar: 50 μm. *P < 0.05, **P < 0.01, ***P < 0.001

Based on the mRNA profiling, we estimated the composition and abundance of immune cells in the lung tissues of non-smokers, smokers, and COPD patients. The results showed that activated NK cells and M2 macrophages were significantly upregulated in COPD, while no significant differences were found in various immune cells among smokers (Fig. 6A). We additionally validated NK cell and M2 macrophage infiltration in the lung tissues of smokers without COPD and smokers with COPD using double immunofluorescence staining (Fig. 6B). In addition, we used the Cmap database to predict prospective small-molecule drugs for the treatment of COPD and demonstrated the 3D structures of the ITK inhibitor and oxybutynin chloride by Pubchem (Additional file 2: Fig. S4).

## Discussion

Generally, COPD is characterized by chronic inflammation, small airway wall remodeling, and lung parenchymal destruction (emphysema). The detailed pathogenesis remains unknown. After more than a decade of development, high-throughput sequencing has infiltrated various fields of life science, not only effectively promoting the development of studies but also gradually being applied to clinical practice. High-throughput sequencing and bioinformatics analysis make it possible to identify a large number of differentially expressed genes, which can be exploited to investigate disease-related genes and biological mechanisms and find potential molecular targets. To the best of our knowledge, there are several published articles exploring multiple kinds of RNA transcripts in COPD. For instance, Liu et al. described 282 mRNAs, 146 lncRNAs, 85 miRNAs, and 81 circRNAs differently expressed in peripheral blood [19]. Feng et al. found 1796 mRNAs, 2207 lncRNAs, and 11 miRNAs differentially expressed in COPD lung tissues [20]. The lung is the primary site of COPD. Sequencing lung tissue can mitigate the effects of comorbidities and give deeper insights into COPD pathogenesis. However, no studies have systematically described and analyzed the expression of mRNAs and non-coding RNAs from the lung tissue of smokers without COPD as well as smokers with COPD, particularly circRNAs. In this study, we were the first to perform whole-transcriptome sequencing on lung tissues from non-smokers, smokers, and COPD patients. We found that there were differences in the expression of 1289 mRNAs, 32 circRNAs, 433 lncRNAs, and 69 miRNAs in smokers and COPD patients, as well as 1561 mRNAs, 33 circRNAs, 545 lncRNAs, and 48 miRNAs in non-smokers and smokers. Meanwhile, in comparison to non-smokers, 379 differentially expressed genes with the same expression trend were also identified in both smokers and COPD patients.

In this study, we discovered 17 hub genes that could increase the risk of COPD in people with a history of smoking and 18 COPD-related hub genes. We also validated some of these hub genes at both RNA and protein levels in human and rat lung tissues. Ribosomal proteins (RPs) are abundant in these hub genes. It has been reported that RP deficiency alters the expression of eight major functional genes, including the cell cycle, cell metabolism, signal transduction, and development [21]. For example, inhibition of RPL13 expression by siRNA in A549 cells can block the cell cycle in the G1 phase, thereby influencing the expression level of CDK2, Cyclin D1, E2F1, p21, and RB1 [22]. When RPL32 is silenced, RPL5 and RPL11 translocate from the nucleolus to the nucleoplasm through ribosomal stress, where they bind to murine double minute 2 and block it from binding to p53, causing p53 to accumulate and inhibiting the proliferation of lung cancer cell lines [23]. Knockdown of RPS9 can significantly inhibit cell proliferation and metastasis and induce apoptosis in NSCLC through Stat3 and Erk signaling pathways [24]. Among these 18 COPD hub genes, only RPL32, RPL13A, and HSP90AA1 have been previously reported in COPD. For instance, Wang et al. revealed that HSP90AA1 is a COPD-related gene and could facilitate squamous cell lung cancer progression [25]. Feng et al. reported that the lung tissue RPL32 and RPL13A are promising diagnostic biomarkers of COPD [20], in agreement with our results. However, most hub genes found in our study have not been reported in COPD. Further studies are needed to reveal the biological effects of these mRNAs with differential expression in COPD.

Subsequently, the biological functions and signaling pathways of differentially expressed mRNAs were determined by GO annotation, KEGG, and GSEA enrichment analysis. The enrichment results suggest that the mitotic spindle, inflammatory response, myc-targets, Wnt/β-catenin signaling, complement, and TGF-β signaling could be associated with the occurrence and development of COPD. It has been reported that TGF-β is vital to the development of M2 macrophages, which are the main culprit in the progression of CS-exposed COPD [26]. Meanwhile, the activation of TGF-β-related pathways may be associated with EMT in both smokers and COPD patients [27]. Our prior research also demonstrated that TGF-β1 induces pSMAD3 expression in BEAS-2B cells, which leads to the EMT transformation of BEAS-2B cells [28]. Previous studies have found that canonical Wnt signaling is vital for the maintenance of stem cell niches in the lung [29]. Increased expression of Wnt5a can activate noncanonical Wnt signaling and contribute to COPD pathogenesis [30]. Specifically, Wnt5a regulates TGF-β1-induced α-smooth muscle actin expression through ROCK-mediated actin polymerization to enhance smooth muscle cell contractility [31]. TGF-β1 is involved in noncanonical Wnt5a signaling and promotes tissue fibrosis by inducing extracellular matrix expression in airway smooth muscle cells [30]. All these studies have shown that TGF-β and Wnt/β-catenin signaling pathways are crucial in COPD pathogenesis.

RBPs can coordinate RNA processing, regulate RNA stability, and affect RNA localization and transport [11]. In our research, the RBP-hub gene network in COPD patients included 130 RBPs, while the RBP-hub gene network shared by smokers and COPD patients included 129 RBPs. Among these RBPs, the previous studies only explored the function of some RBPs, such as METTL14, WTAP, METTL3, ALKBH5, FTO, and so on. METTL14 is a critical mediator of m6A modification that regulates RNA half-life, pre-RNA splicing, processing, and nuclear export [32]. Meanwhile, METTL14/m6A can promote the processing and maturation of pre-miRNAs by interacting with the microprocessor protein DGCR8 [33, 34]. It has been confirmed that RNA methyltransferases such as WTAP and METTL3 and RNA demethylases such as ALKBH5 and FTO are intimately associated with destructive lung diseases, including different types of lung cancer [35, 36]. RBP-driven regulation and therapeutic targeting are being investigated in human lung cancer [37]. However, its role in COPD pathogenesis is less explored. Therefore, further investigation is worthwhile to understand RBP-mediated post-transcriptional regulation of genes in COPD.

Recent research has described the growing importance of ceRNA mechanisms in disease, wherein ncRNA molecules, including lncRNA and circRNA, share a common miRNA response element to regulate cellular processes through complicated RNA networks [19, 38]. Studies have found that the lncRNA PELATON in lung cancer targets the miR-7-5p/CRLS1 axis to inhibit cell proliferation and induce apoptosis [39]. Similarly, the lncRNA PELATON could be utilized as an independent prognostic biomarker for NSCLC patients, and its overexpression could inhibit cell proliferation, migration, and invasion via inhibiting miR-1303 [40]. According to Zhang et al., the lncRNA NORAD promotes NSCLC progression by regulating the miR-26b-5p/COMMD8 axis, providing a potential novel target in NSCLC therapy[41]. LncRNA NORAD also promotes SCLC invasion and EMT by targeting the miR-93-5p/NSE axis [42]. Recently, Han et al. suggested that lncRNA NORAD could be used as a core marker of COPD [43]. However, the functional mechanisms of the circRNA–miRNA–mRNA and lncRNA–miRNA–mRNA networks found in this study have not been reported in COPD. We believe that a systematic and comprehensive investigation of the interaction networks of RNAs with differential expression will provide novel insights into COPD pathogenesis.

We used the CIBERSORTx to investigate the differential levels of immune cell infiltration between smokers and COPD patients and found that the M2 macrophages as well as the activated NK cells were elevated in COPD patients. Macrophages, an important component of the innate immune system, can release cytokines upon stimulation by cigarette smoking or pathogen invasion, leading to the recruitment of neutrophils [44, 45]. These proinflammatory cells can release ROS, proteases, inflammatory cytokines, and chemokines to participate in the degradation of the extracellular matrix, mucus secretion, and cell injury, thereby promoting the progression of emphysema [2, 46]. It has been found that CS can induce the polarization of macrophages to an M2 phenotype and produce anti-inflammatory cytokines [47]. Similar to our study, Liu et al. found that the M2 phenotype is dominant in COPD patients, CS-exposed mice, and macrophages treated with cigarette smoke extract [44]. NK cells are important innate immune effectors, and their abnormal activation can lead to tissue injury and chronic inflammation [48]. CS exposure may aggravate the injury of lung epithelial cells by increasing the cytotoxicity and cytokine production of NK cells [49]. Overall, the M2 macrophages and the activated NK cells play a pivotal role in airway inflammation and lung parenchyma destruction.

Finally, according to the differentially expressed mRNAs, we used the Cmap to explore potential therapeutic drugs for COPD. The two drugs with higher negative connection scores were the ITK inhibitor and oxybutynin chloride. ITK is critical in the T cell receptor signaling pathway, regulating the expression of transcription factors such as NF-AT and NF-κB and proinflammatory cytokines from Th2 cells, Th17 cells, and mast cells [50]. Studies have found that the ITK inhibitor can reverse the changes in airway inflammation and Th17 cell/oxidative stress in mice with acute lung injury [51]. Nadeem et al. found that the ITK inhibitor significantly inhibits Th17/Th2 and neutrophilic/eosinophilic airway inflammation in asthmatic mice and concluded that the ITK inhibitor could be used as an alternative therapeutic option for corticosteroid-refractory asthma [52]. Oxybutynin chloride is the chloride salt form of oxybutynin, an anticholinergic agent with antispasmodic activity. Currently, studies on oxybutynin chloride in the respiratory system have mainly focused on the treatment of obstructive sleep apnea [53–55]. We look forward to future clinical trials to evaluate the feasibility of the ITK inhibitor and oxybutynin chloride for the treatment of COPD.

Nevertheless, there are some limitations to our study. Since all patients are from the local hospital, the results of the study may have certain regional limitations. Additionally, the biological regulatory networks at the post-transcriptional level and COPD therapeutic drugs are based on only bioinformatics predictions and will require further in-depth investigations with in vivo and in vitro experiments in the future. We are currently devoting significant efforts toward investigating these potential functions and mechanisms in cell culture and animal experiments.

## Conclusions

In summary, our study reveals potentially significant hub genes, RNAs, pathways, and biological regulatory networks associated with COPD, particularly the RBP-hub gene and circRNA/lncRNA–miRNA–mRNA networks, and potential therapeutic drugs, providing a novel perspective on the pathogenesis and therapeutic strategies of COPD. So far, very few studies have focused on multiple kinds of RNAs with differential expression in COPD, so this study offers an important RNA resource for further studies of COPD in the future.

### Supplementary Information

**Additional file 1: Table S1**. Primers sequences for validation of hub genes. **Table S2**. Differentially expressed mRNAs in non-smokers and smokers. **Table S3**. Differentially expressed circRNAs in non-smokers and smokers. **Table S4**. Differentially expressed lncRNAs in non-smokers and smokers. **Table S5**. Differentially expressed miRNAs in non-smokers and smokers. **Table S6**. Differentially expressed mRNAs in smokers and COPD patients. **Table S7**. Differentially expressed circRNAs in smokers and COPD patients. **Table S8**. Differentially expressed lncRNAs in smokers and COPD patients. **Table S9**. Differentially expressed miRNAs in smokers and COPD patients. **Table S10**. GSEA enrichment analysis of gene expression data in smokers and COPD patients.

**Additional file 2**: **Fig. S1. **GO_BP annotation and KEGG enrichment analysis of differentially expressed mRNAs shared by smokers and COPD patients. **Fig. S2.** Pathway and process enrichment analysis of hub genes shared by smokers and COPD patients. **Fig. S3.** (A) Differentially expressed circRNA-miRNA-mRNA network in COPD patients. (B) Differentially expressed circRNA-miRNA-mRNA network shared by smokers and COPD patients. **Fig. S4.** The 3D structures of the ITK inhibitor and oxybutynin chloride.

## Data Availability

The datasets generated or analyzed during this study are included in the article and its Additional files. Additionally, all data from this study can be obtained from the authors upon request.

## Abbreviations

BALF: Bronchoalveolar lavage fluid
Cchord: Chord compliance
Cdyn: Dynamic compliance
ceRNA: Competing endogenous RNA
COPD: Chronic obstructive pulmonary disease
CS: Cigarette smoke
EMT: Epithelial–mesenchymal transition
FRC: Functional residual capacity
MAN: Mean alveolar number
MLI: Mean linear intercept
RBP: RNA-binding protein
RI: Resistance
RPs: Ribosomal proteins
TLC: Total lung capacity
