# Supplementary material for: Systematic analysis of various RNA transcripts and construction of biological regulatory networks at the post-transcriptional level for chronic obstructive pulmonary disease
Source: J Transl Med. 2023 Nov 7;21:790. doi: 10.1186/s12967-023-04674-7 (PMC10631086; doi:10.1186/s12967-023-04674-7)
Supplement: Supplementary file 2 — Additional file 2: Fig. S1. GO_BP annotation and KEGG enrichment analysis of differentially expressed mRNAs shared by smokers and COPD patients. Fig. S2. Pathway and process enrichment analysis of hub genes shared by smokers and COPD patients. Fig. S3. (A) Differentially expressed circRNA-miRNA-mRNA network in COPD patients. (B) Differentially expressed circRNA-miRNA-mRNA network shared by smokers and COPD patients. Fig. S4. The 3D structures of the ITK inhibitor and oxybutynin chloride. [file 12967_2023_4674_MOESM2_ESM.doc]

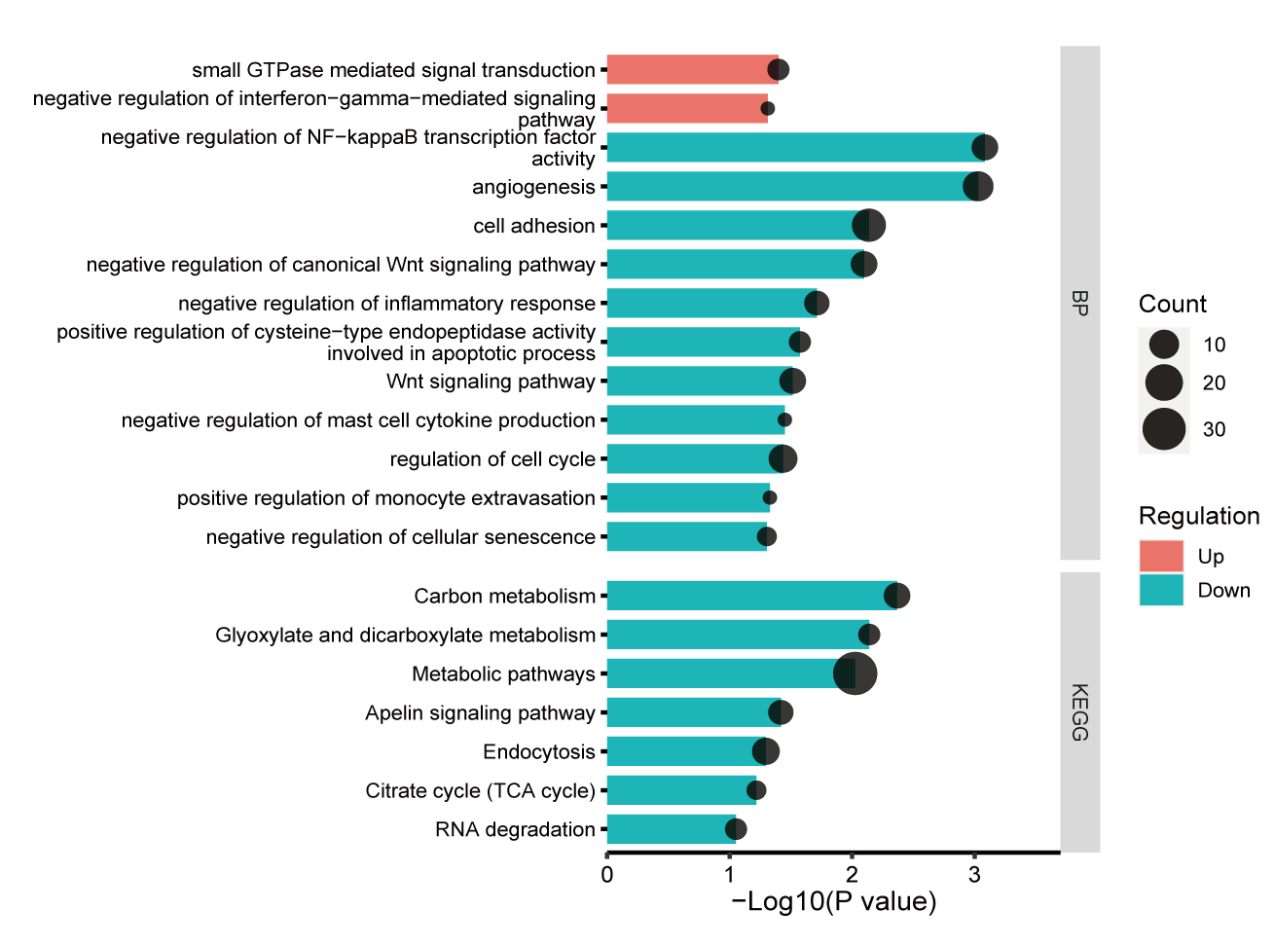


**Fig. S1** GO_BP annotation and KEGG enrichment analysis of differentially expressed mRNAs shared by smokers and COPD patients.


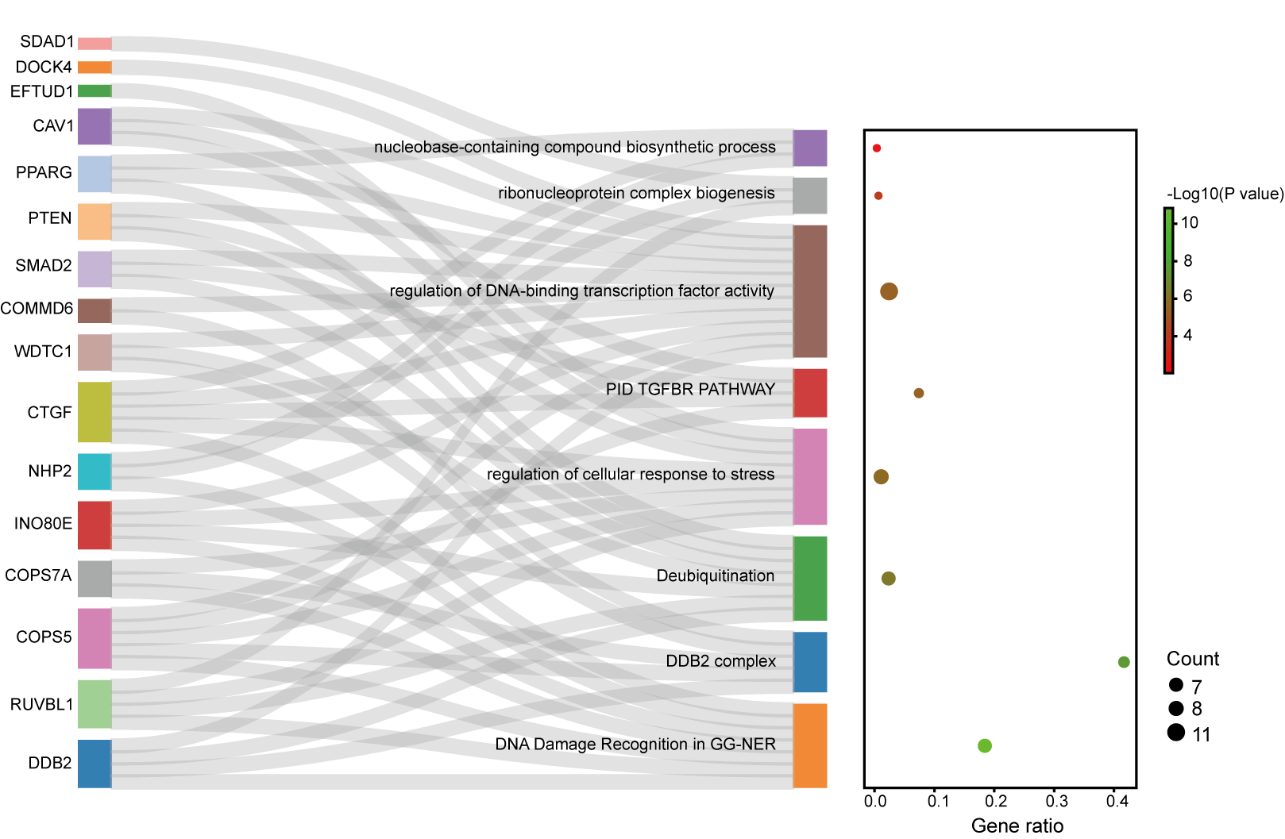


**Fig. S2** Pathway and process enrichment analysis of hub genes shared by smokers and COPD patients


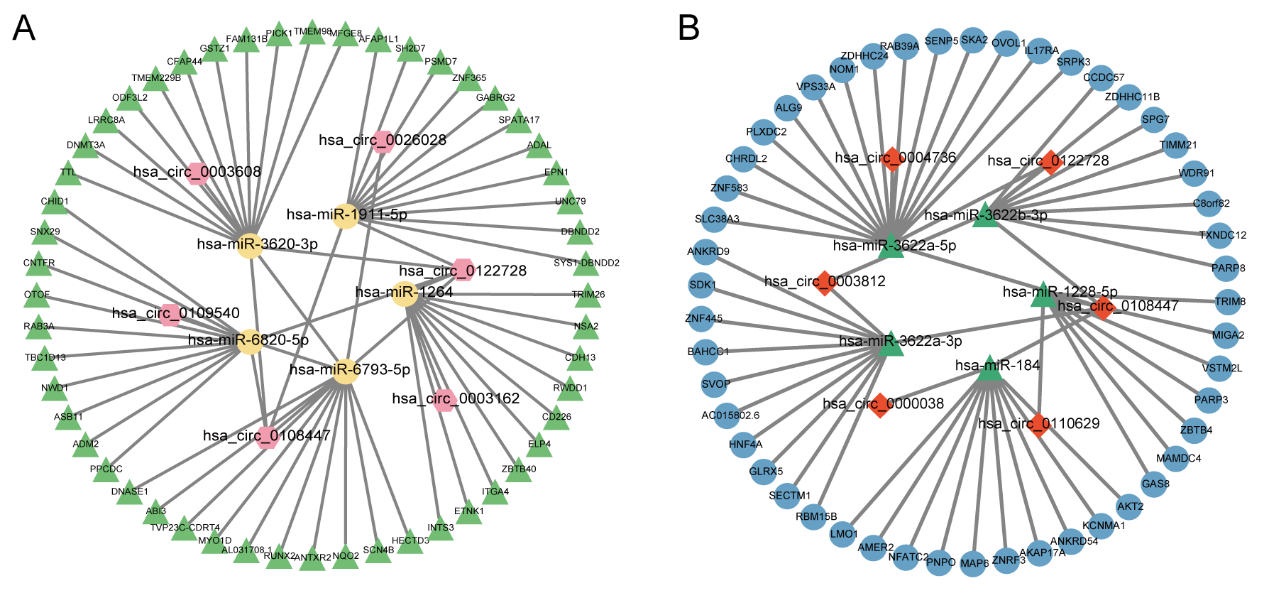


**Fig. S3** (A) Differentially expressed circRNA-miRNA-mRNA network in COPD patients. (B) Differentially expressed circRNA-miRNA-mRNA network shared by smokers and COPD patients.


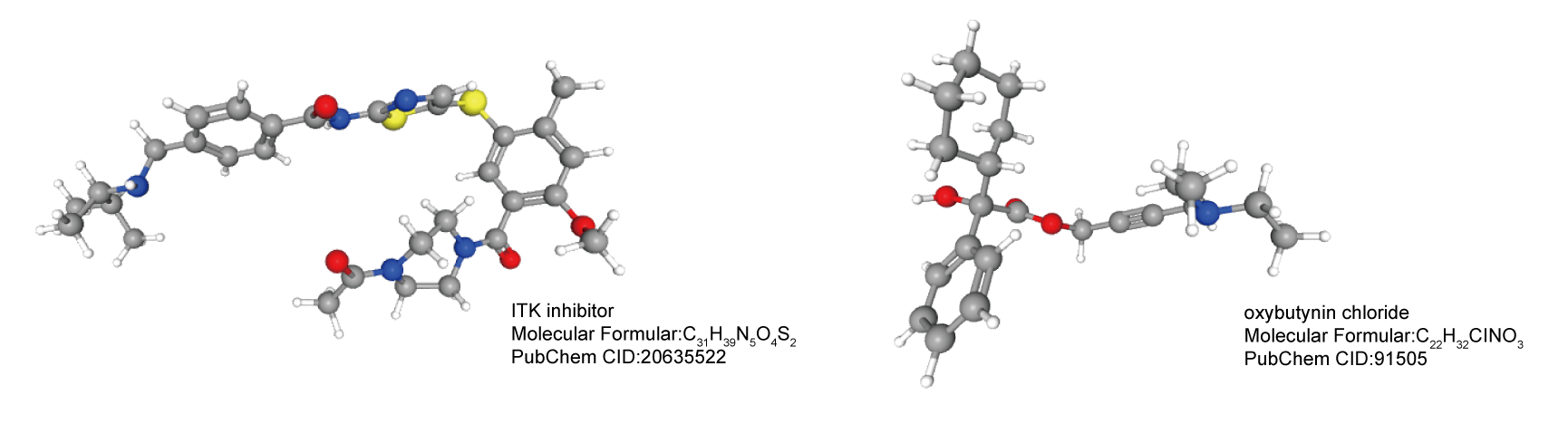


**Fig. S4** The 3D structures of the ITK inhibitor and oxybutynin chloride.

Raw data for western blot


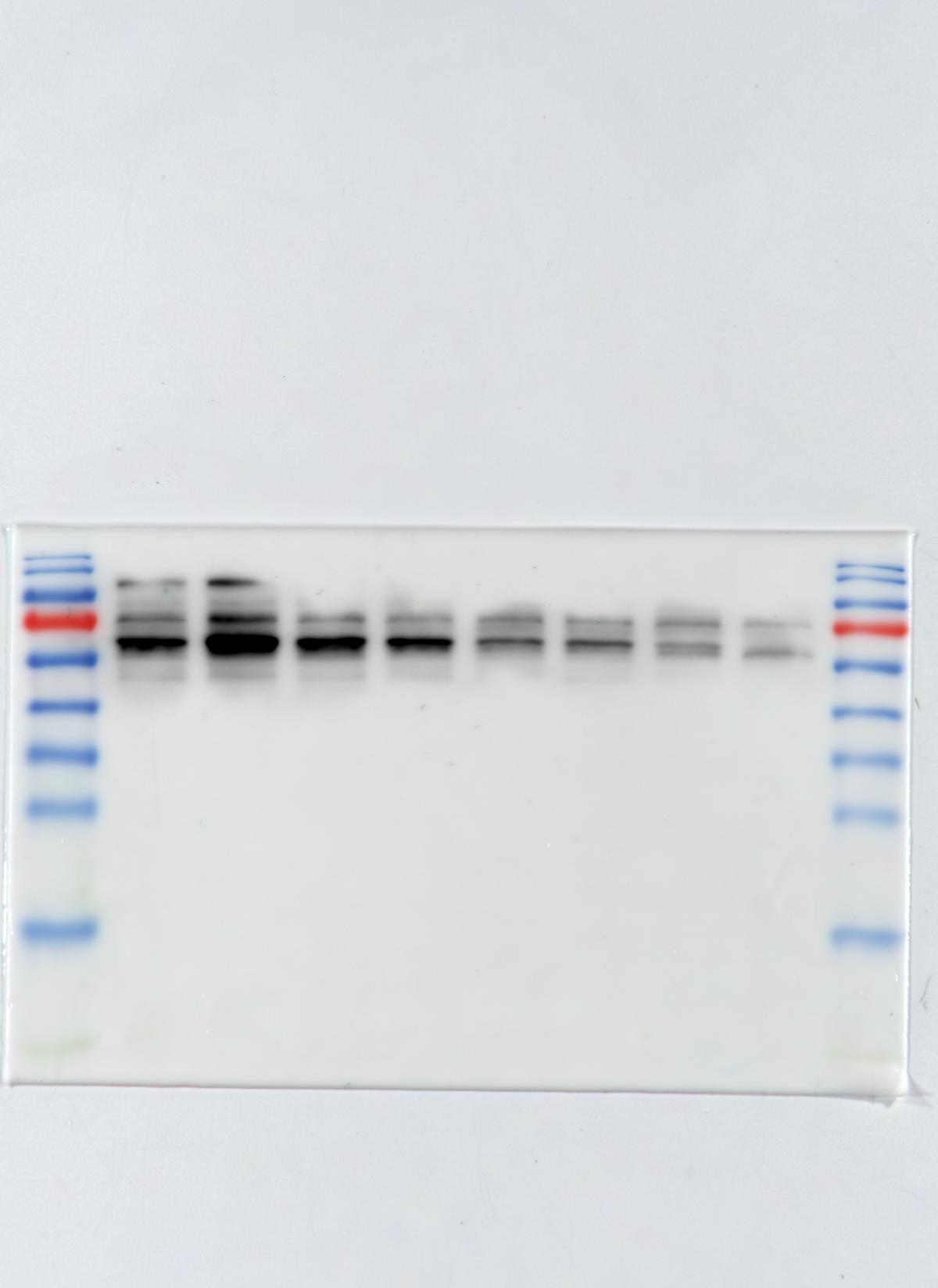

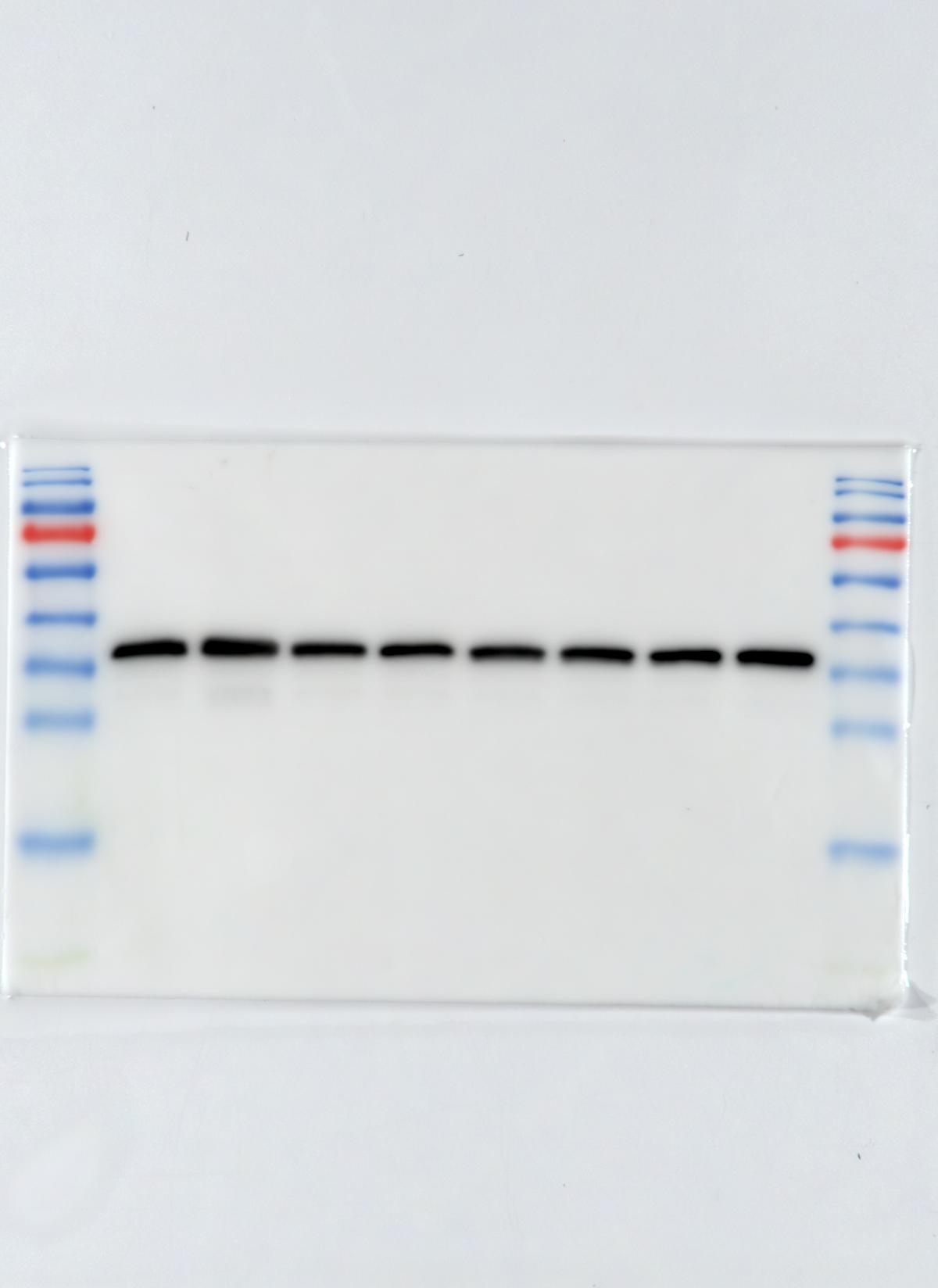


Western blot analysis shows DDB2 (left panel) and GAPDH (right panel) protein expression in lung tissue from COPD and control rats.


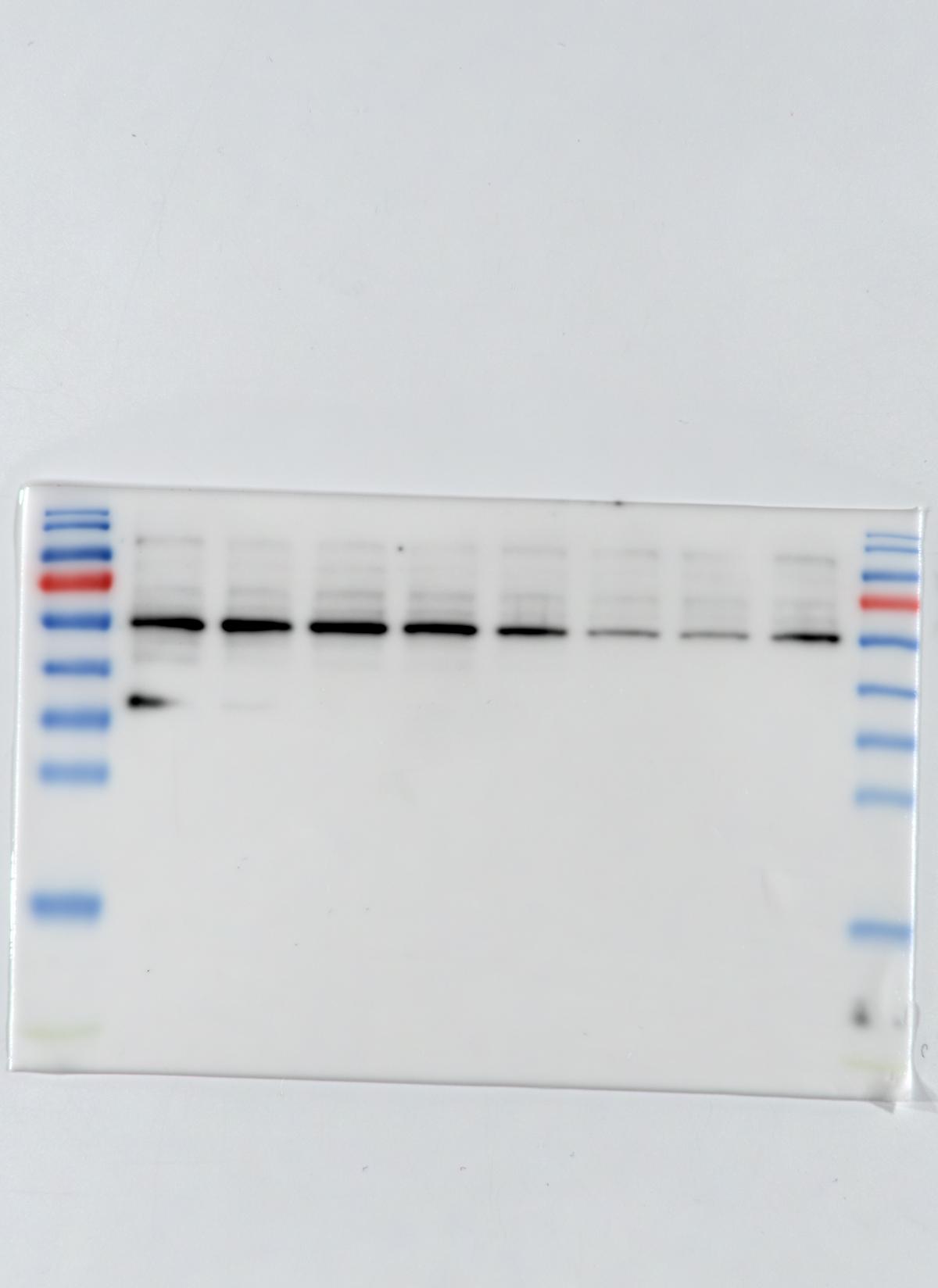

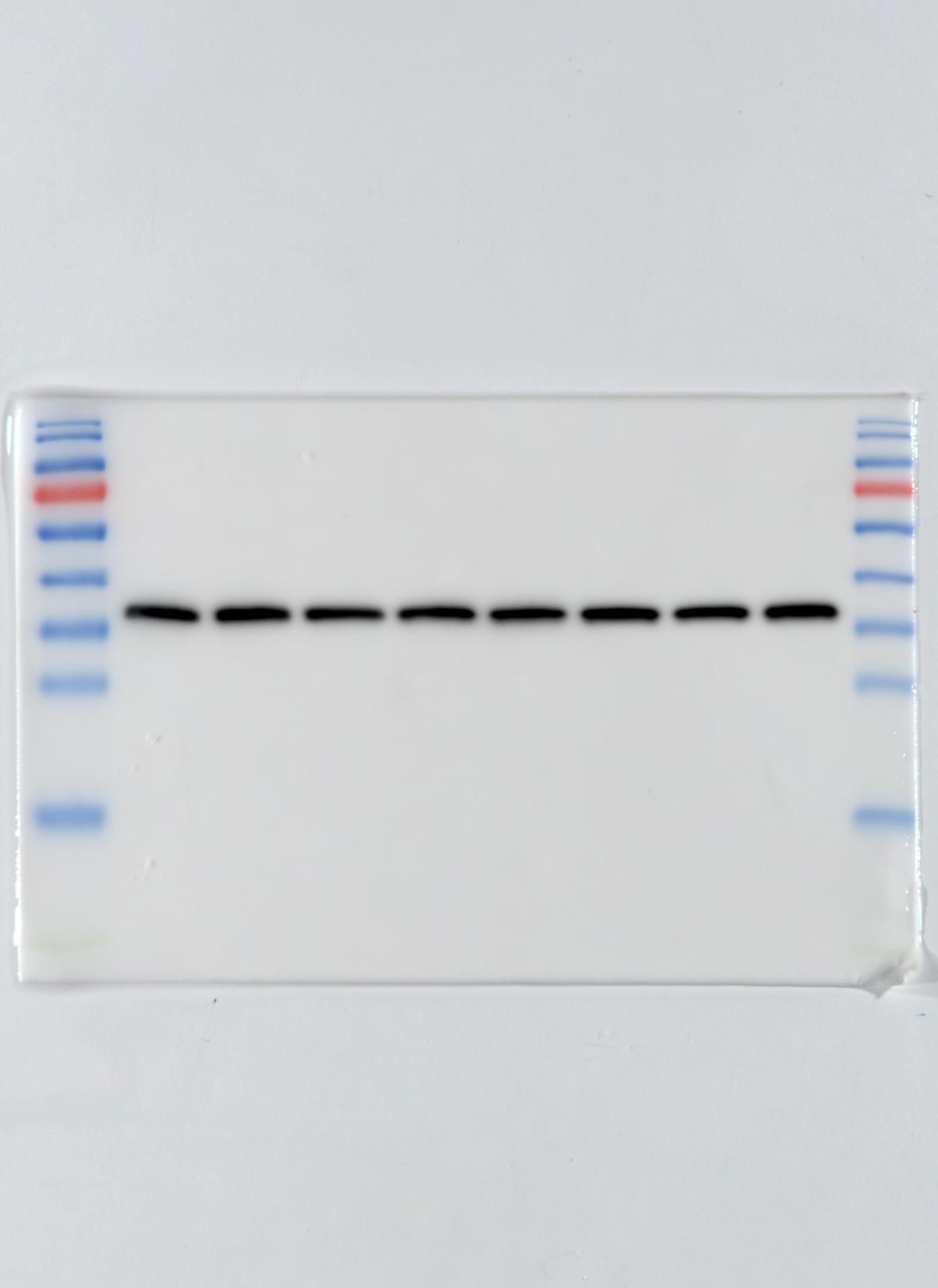


Western blot analysis shows RUVBL1 (left panel) and GAPDH (right panel) protein expression in lung tissue from COPD and control rats.


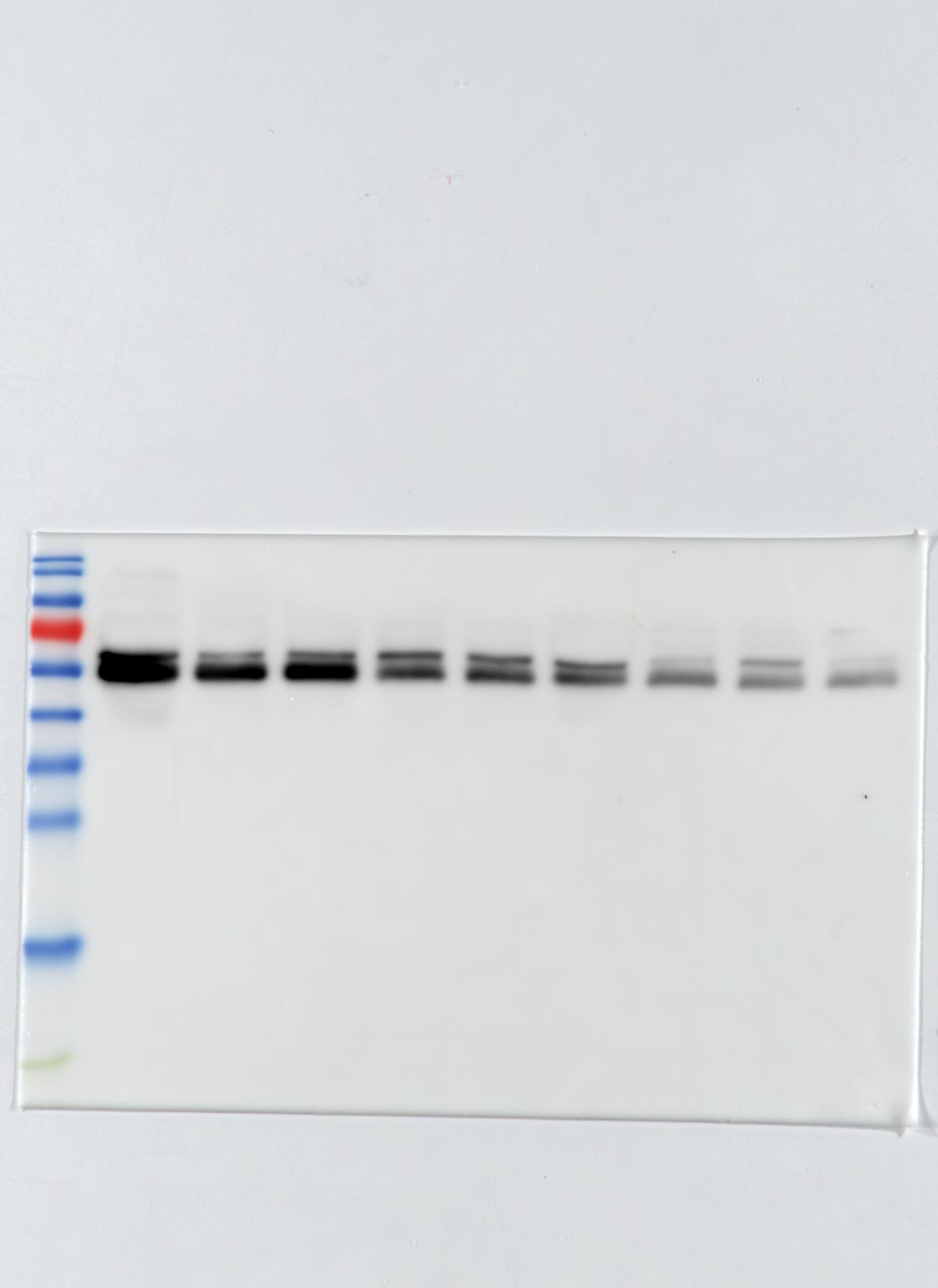

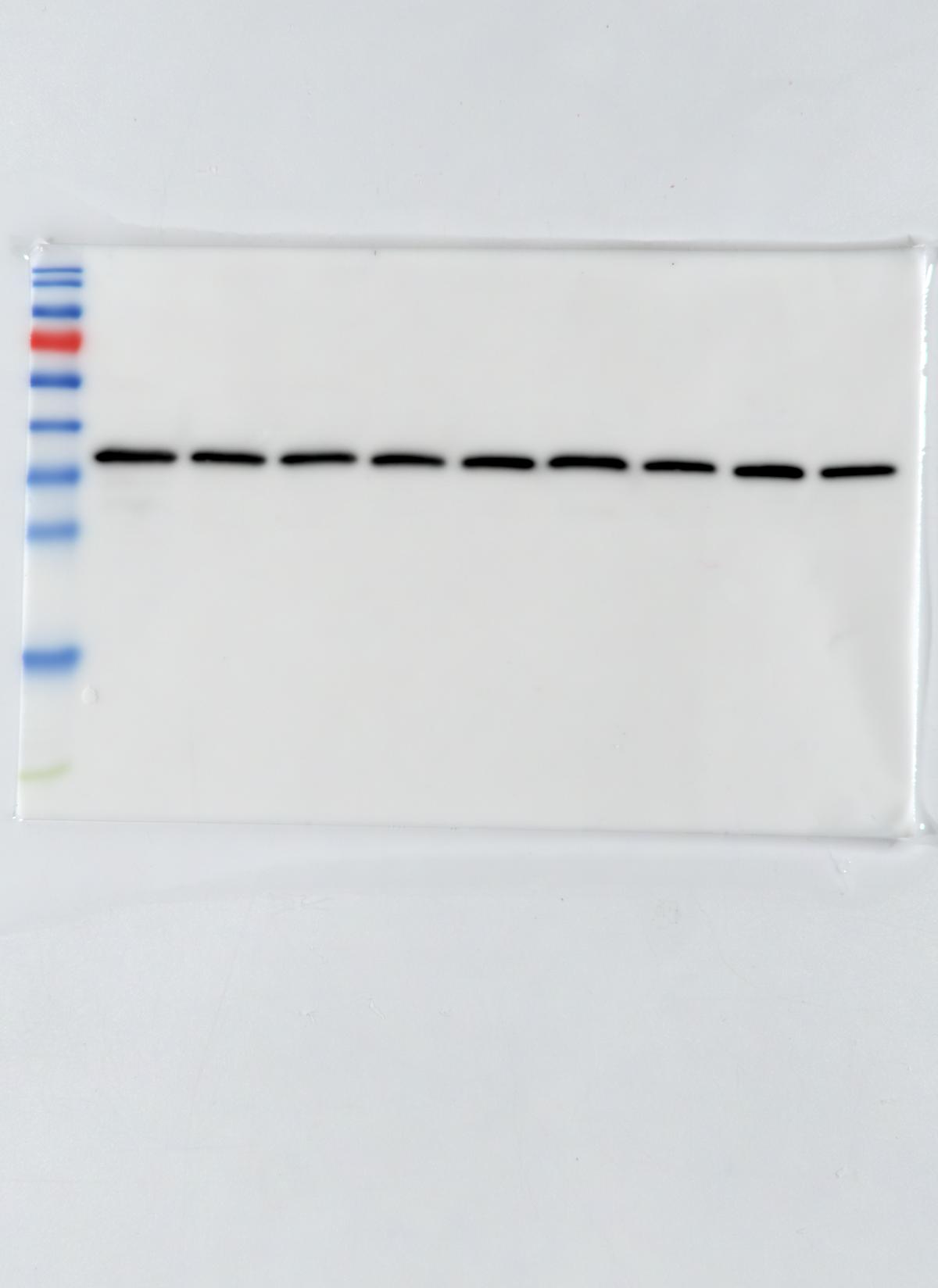


Western blot analysis shows DDB2 (left panel) and GAPDH (right panel) protein expression in lung tissue from non-smokers, smokers without COPD, and smokers with COPD.


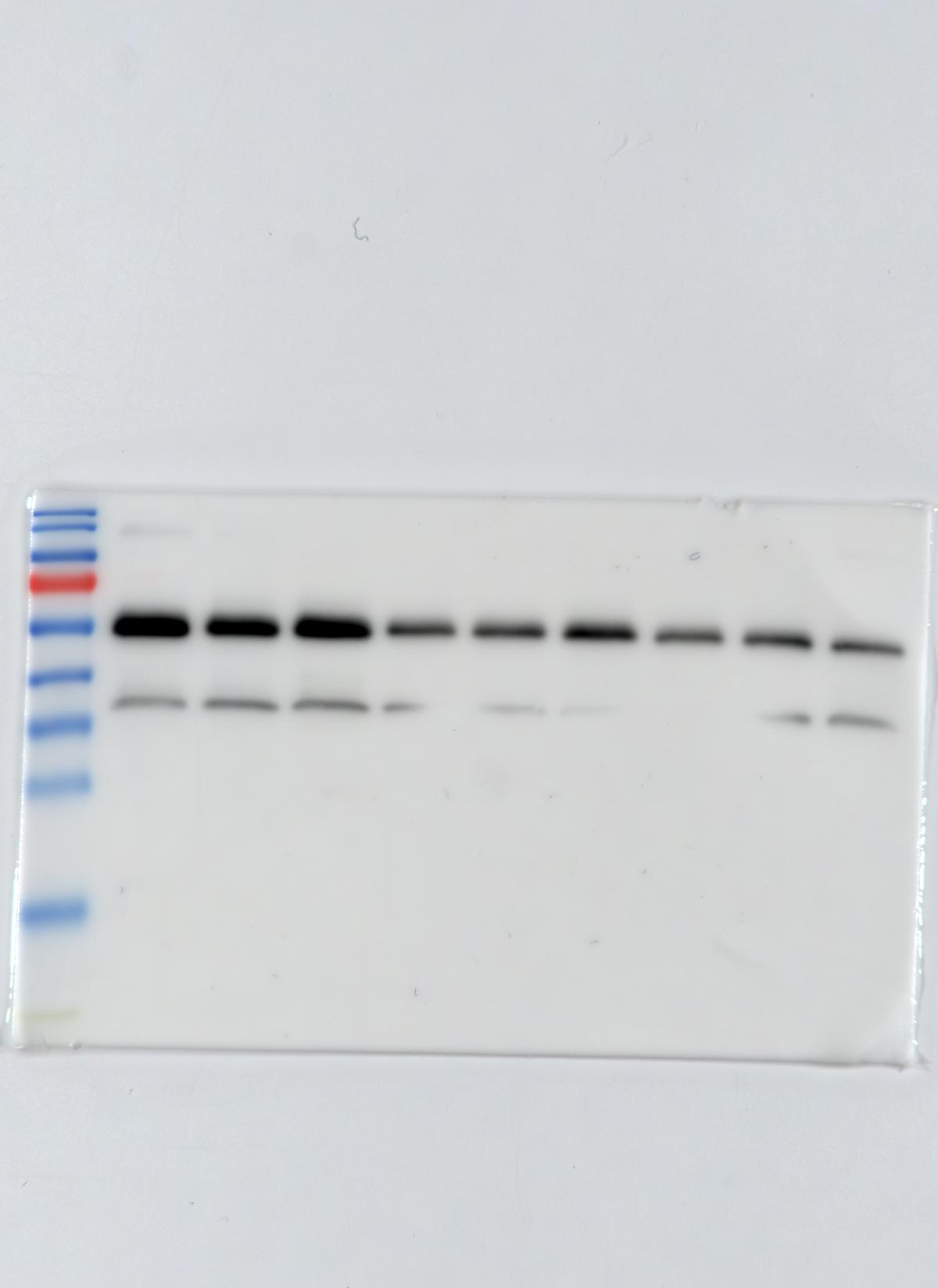

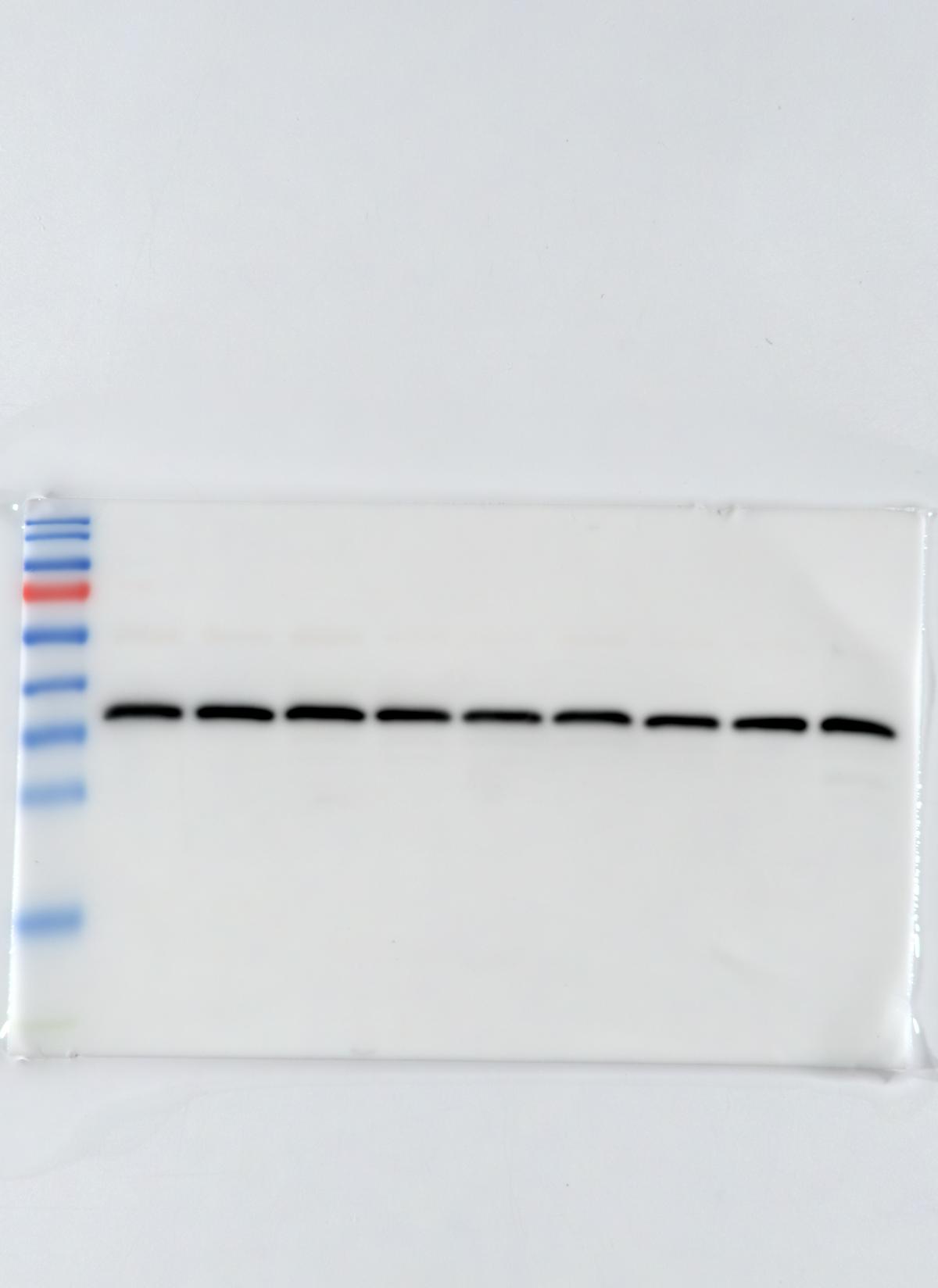


Western blot analysis shows RUVBL1 (left panel) and GAPDH (right panel) protein expression in lung tissue from non-smokers, smokers without COPD, and smokers with COPD.
